# Apoptosis-like cell death in *Leishmania donovani* treated with KalsomeTM10, a new liposomal amphotericin B

**DOI:** 10.1371/journal.pone.0171306

**Published:** 2017-02-07

**Authors:** Md. Shadab, Baijayanti Jha, Mohammad Asad, Makaraju Deepthi, Mohd. Kamran, Nahid Ali

**Affiliations:** Infectious Diseases and Immunology Division, Indian Institute of Chemical Biology, Jadavpur, Kolkata, West Bengal, India; Meharry Medical College, UNITED STATES

## Abstract

**Objective:**

The present study aimed to elucidate the cell death mechanism in *Leishmania donovani* upon treatment with KalsomeTM10, a new liposomal amphotericin B.

**Methodology/Principal findings:**

We studied morphological alterations in promastigotes through phase contrast and scanning electron microscopy. Phosphatidylserine (PS) exposure, loss of mitochondrial membrane potential and disruption of mitochondrial integrity was determined by flow cytometry using annexinV-FITC, JC-1 and mitotraker, respectively. For analysing oxidative stress, generation of H_2_O_2_ (bioluminescence kit) and mitochondrial superoxide O_2_^−^ (mitosox) were measured. DNA fragmentation was evaluated using terminal deoxyribonucleotidyl transferase mediated dUTP nick-end labelling (TUNEL) and DNA laddering assay. We found that KalsomeTM10 is more effective then Ambisome against the promastigote as well as intracellular amastigote forms. The mechanistic study showed that KalsomeTM10 induced several morphological alterations in promastigotes typical of apoptosis. KalsomeTM10 treatment showed a dose- and time-dependent exposure of PS in promastigotes. Further, study on mitochondrial pathway revealed loss of mitochondrial membrane potential as well as disruption in mitochondrial integrity with depletion of intracellular pool of ATP. KalsomeTM10 treated promastigotes showed increased ROS production, diminished GSH levels and increased caspase-like activity. DNA fragmentation and cell cycle arrest was observed in KalsomeTM10 treated promastigotes. Apoptotic DNA fragmentation was also observed in KalsomeTM10 treated intracellular amastigotes. KalsomeTM10 induced generation of ROS and nitric oxide leads to the killing of the intracellular parasites. Moreover, endocytosis is indispensable for KalsomeTM10 mediated anti-leishmanial effect in host macrophage.

**Conclusions:**

KalsomeTM10 induces apoptotic-like cell death in *L*. *donovani* parasites to exhibit its anti-leishmanial function.

## Introduction

Leishmaniasis, a vector borne parasitic disease, is prevalent in 98 countries with 350 million people at a risk of infection [1]. Disease manifestations include visceral, cutaneous and mucocutaneous forms. Visceral leishmaniasis (VL, also known as kala-azar), caused by *L*. *donovani* and *L*. *infantum* in Old World and *L*. *chagasi* in New World, is often deadly if left untreated [2]. Currently, there is no anti-leishmanial vaccine and control measures rely on few conventional drugs. Pentavalent antimonials that have been the torch bearers in the treatment of VL are not free from toxicity (nephro- and hepato-), and associated side effects of long term intravenous injections. Furthermore, the emergence of resistant parasites has worsened the scenario of VL therapy [3]. Amphotericin B, a polyene macrolide, is the best drug available for the treatment of kala-azar and is effective in curing patients also infected with antimony resistant parasites. However, it remains a second-line drug due to its severe toxicities. Moreover, since the drug is administered parenterally through long term hospitalization the overall cost of treatment increases. Hence, to ameliorate these problems, lipid formulations of amphotericin B including liposomal amphotericin B [L-AmB (Ambisome)], colloidal dispersion of amphotericin B [ABCD (Amphotec)] and amphotericin B lipid complex [ABLC (Abelcet)] were developed [4]. These lipid formulations offered a much higher treatment efficacy with comparatively shorter duration of administration, reducing the cost of hospitalization significantly. However, one of the major drawbacks associated with these formulations is that they entrap small amounts of amphotericin B, thereby increasing the dose of administration for efficient cure. This in turn not only increases the cost as amphotericin B is itself quite costly, but also increases the risk of lipid associated side effects. Moreover, treatment failure of Ambisome in AIDS patients co-infected with VL (by *L*. *infantum*) in Europe [5], in below 5 years aged children infected with Brazilian VL (due to *L*. *chagasi*), in Indian kala-azar [6] and recently in Sudanese patients of VL is worrisome [7]. Therefore, new lipid formulations of amphotericin B are in need to overcome these drawbacks. To address some of these issues, Kalsome TM10, a new liposomal formulation of amphotericin B has recently been developed [8]. It offers an advanced alternative option wherein the drug is packaged with ergosterol, in place of cholesterol, which provides a much greater binding affinity (nearly > 8 folds) to amphotericin B and thus facilitates its higher loading in a smaller amount of liposome. This reduces not only the toxicity but also the dose of the drug as well as the amount of liposome required for entrapping the drug, thereby lowering the overall cost significantly.

The in vivo toxicity and anti-leishmanial efficacy of Kalsome TM10 have already been tested and the results showed that the toxicity of amphotericin B was almost negligible, when the drug was used in the liposomal form, compared to the deoxycholate form (Fungizone) [9]. Moreover, Kalsome TM10 showed almost complete clearance of parasites in infected BALB/c mice, which was correlated with enhanced Th1 response and suppressed Th2 response [8]. Taken together, these results confirm that Kalsome TM10 is efficacious against in vivo model of murine VL with no major toxic effects. However, the actual mechanism of action of KalsomeTM10 on *Leishmania* parasites is yet not clear. Given the recent emergence of amphotericin B resistant parasites [10], it would be of interest to investigate the relevant cell death mechanism in KalsomeTM10 treated *L*. *donovani*, so that these could be used as a tool to estimate the changing susceptibility of these parasites to amphotericin B and its liposomal formulations. The information gained from the mechanistic study of killing would allow development of effective anti-leishmanial interventions against the parasites and those showing a kind of resistance to amphotericin B.

In this study we did not used empty liposome owing to its no activity in vivo model of VL [9]. We showed here that KalsomeTM10 induces effective clearance of parasites in infected host macrophages at doses significantly lower than that induced by amphotericin B and Ambisome. In addition, we showed that the leishmanicidal effect of KalsomeTM10 is a consequence of induction of apoptotic-like cell death in promastigotes as well as intracellular amastigotes. We report that the KalsomeTM10 treated parasites demonstrated numerous cytoplasmic, nuclear, and membrane features that resembles to that of the apoptotic metazoan cells. These include cell shrinkage, DNA fragmentation into oligonucleosome-sized fragments, phosphatidylserine exposure and mitochondrial dysfunction etc. All the above features are the characteristic events of the cells in the early phase of apoptosis. All together, our results show that KalsomeTM10 induces apoptotic-like cell death in *L*. *donovani* parasites.

## Materials and methods

### Ethics statement

The study was approved by and carried out under the guidelines of the Ethical Committee of the Indian Institute of Chemical Biology, Kolkata. All subjects who participated in this study provided informed consent in writing according to the Indian Institute of Chemical Biology guidelines and approval. The animal experiments were approved by the Animal Ethical Committee (147/1999/CPSCEA) of the institute, according to the National Regulatory Guidelines issued by the Committee for the Purpose of Control and Supervision on Experimental Animals (CPCSEA), under the Division of Animal Welfare, Ministry of Environment and Forest, Government of India.

### Animals and parasite

BALB/c mice, bred in the animal house facility of Indian Institute of Chemical Biology (Calcutta, India), were used for the experiments. For parasite maintenance the *L*. *donovani* strain AG83 (MHOM/IN/1983/AG83), originally isolated from an Indian kala-azar patient was periodically injected in appropriate numbers in 4–6 weeks old Syrian golden hamsters (*Mesocricetus auratus*) reared in pathogen-free animal care facility of the Indian Institute of Chemical Biology. The infected hamsters were sacrificed at two months infection to isolate *L*. *donovani* amastigotes from the spleens. These amastigotes were transformed into promastigotes in M199 supplemented with 10% FCS, 2 mM glutamine, penicillin G (100 U/ml) and streptomycin sulfate (100 ug/ml) at 22°C.

### Reagents

RPMI 1640, M199, penicillin, streptomycin, PEG-Catalase (PEG-Cat) and PEG-Superoxide dismutase (PEG-SOD) were purchased from Sigma Chemical Co. (St Louis, MO, USA). Fetal calf serum (FCS) was from (GIBCO/BRL, Grand Island, NY). *N*-Acetyl-L-cysteine (NAC) and NG-monomethyl L-arginine (L-NMMA) was purchased from Merck (GmBH, Germany). MitoTracker deep red and MitoSOX^™^ red were obtained from Molecular Probes (Eugene, OR, USA). The new formulation of liposomal amphotericin B, Kalsome^™^10, was a kind gift from Lifecare Innovations Pvt. Ltd. Gurgaon, India. Conventional liposomal formulation of AmB (AmBisome) was purchased from Gilead Science, Inc., (USA). Amphotericin B deoxycholate was purchased from Bharat Serums and Vaccines Limited (India).

### Formulation of KalsomeTM10 and Ambisome

KalsomeTM10 is a sterol enriched, mixed lamellarity, amphotericin B intercalating liposome in 0.9% saline, wherein, the concentration of amphotericin B is 0.2 mg/mg total lipid. Ergosterol constitutes almost 50% of the total liposomal lipid, overall consisting of phospatidylcholine, ergosterol and AmB in 5:2:1.8 molar ratios [8].

Ambisome is unilamellar bilayer liposome with amphotericin B intercalated within the membrane wherein, the concentration of amphotericin B is 0.143 mg/mg total lipid [11].

### Nanoparticle Tracking Analysis (NTA)

NTA measurements were done with a NanoSight LM20 (NanoSight, Amesbury, United Kingdom), according to the recommended protocol using appropriate dilutions of KalsomeTM10 and Ambisome. The software used for capturing and analyzing the data was the NTA 2.0 Build 127. The mean size and SD values were obtained using the NTA software. These values correspond to the arithmetic values calculated with the sizes of all the particles analyzed by the software.

### Cell viability assay

AlamarBlue^®^ reagent (Life Technologies, Carlsbad, USA) was employed for *L*. *donovani* promastigote and mouse peritoneal macrophage cell viability assay. Live cells maintain a reducing environment within their cytosol. AlamarBlue^®^ reagent contain Resazurin/AlamarBlueH (7-Hydroxy-3H-phenoxazin-3- one 10-oxide) as an active ingredient which is non-toxic and cell permeable. This active compound is blue in colour and virtually non-fluorescent. Upon entering cells, resazurin is reduced to resorufin which is red in colour and highly fluorescent. Hence, viable cells continuously convert resazurin to resorufin thereby increasing the overall fluorescence and colour of the media surrounding the cells. Assays were performed in sterile 96-well plates using 100 μl of late log-phase promastigotes adjusted to (1x10^7^ cells/ml) and 5x10^5^ macrophages in 100 μl of medium. These cells were incubated in the absence (control) or presence of varying concentrations of KalsomeTM10, Ambisome and amphotericin B for different time periods. After the completion of treatment, 10 μl of the resazurin dye (0.01%) was added to these cells, and were incubated for a further 4 h at 37°C. We have seen that culturing the promastigotes at 37°C for 4 h does not affect the viability of the parasites as also shown by an earlier report [12]. Cells were then analyzed in a MicroQuant microplate reader (Biotek-Instrument Inc., Winooski, VT, USA) at a wavelength of 570 nm, using 600 nm as reference wavelength (normalized to the 600 nm value). Cell viability was evaluated based on comparison with untreated control cells. ED_50_ and ED_90_ defined as the KalsomeTM10 concentrations that inhibited resazurin reduction with respect to control parasites by 50% and 90%, respectively, were calculated using sigmoidal regression analysis (Microsoft Excel).

### Determination of cellular morphology in *L*. *donovani* promastigotes

*L*. *donovani* promastigotes (1×10^7^ cells/ml) were treated with different concentrations of KalsomeTM10, and incubated at 22±2°C for 4 h. The cells were then harvested, resuspended in PBS (pH 7.4), and observed under optical microscope (Laika DMI 4000, Leica and Zeiss Co., Cambridge, England). The cellular morphology was recorded through QWin software and images were processed using Adobe Photoshop 5.5 (Adobe Systems, Inc., Mountain View, CA, USA) software. For scanning electron microscopy KalsomeTM10 treated and untreated cells were first quick-fixed in 2.5% glutaraldehyde and then post-fixed with 1% OsO4. These processed cells were gradually dehydrated and air-dried. They were finally sputter-coated with gold palladium and examined in a JEOL JSM-5200 electron microscope using an accelerating voltage of 20 kV.

### Evaluation of Annexin V-fluorescein isothiocyanate (FITC)/propidium iodide (PI) binding of cells through flow cytometry

Binding of Annexin V-FITC/PI to cells was assessed using FITC Annexin V Apoptosis Detection Kit II (BD Pharmingen^™^, New Jersey, USA) according to the manufacturer’s instructions. Briefly, 100 μl of promastigotes (1x10^7^/ml) incubated with varying concentrations of KalsomeTM10, Ambisome and amphotericin B for different time periods were harvested and washed twice with cold PBS. Then they were resuspended in 100 μl binding buffer and transferred to a 5 ml culture tube. 5 μl of FITC Annexin V was added and the cells were incubated for 15 min at room temperature (25°C) in the dark. 400 μl of binding buffer was further added to each tube along with 5 μl PI. The cells were then analysed on a flow cytometer (Becton Dickinson, LSR Fortessa, New Jersey, USA) using FACS Diva software (BD Biosciences, New Jersey, USA).

### Measurement of mitochondrial membrane potential (Δ*ѱ*_m_) and mitochondrial integrity

Mitochondrial membrane potential (Δ*ѱ*_m_) was measured using the BD^™^ MitoScreen flow cytometry mitochondrial membrane potential detection kit, according to the manufacturer’s instructions. Briefly, 100μl of KalsomeTM10-treated and non treated *L*. *donovani* promastigotes (1x10^7^/ml) were collected and centrifuged at 400 × *g* for 5 minutes at room temperature. Supernatant was then carefully removed and the cells were resuspended in 0.5 ml of freshly prepared working solution of JC-1 dye and incubated for 10–15 min at 37°C in a CO_2_ incubator. Subsequently, the stained cells were washed twice and resuspended in 0.5 ml of assay buffer for analysis by flow cytometry in Becton Dickinson, LSR Fortessa using FACS Diva software.

### Evaluation of intracellular ATP depletion

The depletion of the intracellular ATP pool in KalsomeTM10 treated and untreated cells was measured using the ATP determination Kit (Molecular Probes) according to the manufacturer's instructions. Briefly, the cells were washed and then resuspended in PBS. To this cell suspension, 9 volumes of boiling 100 mM Tris, 4 mM EDTA pH 7.75 was added and further incubated for 2 min at 100°C. Cells were then centrifuged and the supernatants transferred to a fresh tube on ice until measurement. The sample lysate was then mixed with the reaction buffer provided in the kit and the concentration of ATP was determined using ATP standard curve generated from the readings on Synergy H1 Hybrid reader (BioTek).

### Determination of intracellular reactive oxygen species (ROS) generation

Intracellular H_2_O_2_ levels in treated and untreated cells were measured using the ROS-Glo assay kit (Promega, Madison, WI, USA) according to the manufacturer's instructions. The KalsomeTM10 treated and untreated cells were added to an opaque white 96 well plate in RPMI media. The H_2_O_2_ substrate solution was then added, making the final volume to 100 μl. The plate was then incubated at 37°C in a 5% CO_2_ incubator for 60 min. 100 μl of the ROS-Glo detection solution was then added to each well. After additional 20 min incubation at room temperature, bioluminescent readings were obtained using a Synergy H1 Hybrid reader (BioTek). Mitochondrial superoxide in KalsomeTM10 treated and untreated cells was measured using MitoSOX^™^ red and analysed on a flow cytometer (Becton Dickinson, LSR Fortessa) with FACS Diva software.

### Determination of intracellular glutathione (GSH) content and lipid peroxidation products

Intracellular GSH content in treated and untreated cells was measured using the Glutathione Fluorometric Assay Kit (Biovision, USA) according to the manufacturer's instructions. Briefly, the sample lysate was mixed with the reaction buffer provided in the kit and the concentration of GSH was calculated from the GSH standard curve generated using the readings on Synergy H1 Hybrid reader (BioTek). For the estimation of lipid peroxidation products, the treated and untreated cells were washed twice with PBS and suspended in 1 ml of 15% sodium dodecyl sulfate (SDS)-PBS solution. The fluorescence intensities of the total fluorescent lipid peroxidation products was then obtained [13] through readings on Synergy H1 Hybrid reader (BioTek) with excitation at 360 nm and emission at 430 nm.

### Evaluation of in vivo caspase-like activity

In vivo caspase-like activity in KalsomeTM10, Ambisome and amphotericin B treated and untreated cells were determined using Intracellular Caspase Detection ApoStat kit (R&D Systems, Inc., Minneapolis, USA) according to the manufacturer's instructions. Briefly, cells were stained directly during the last 30 minutes of the apoptosis induction period. For this, 1 μl of ApoStat per 100 μl of culture volume was added. The staining was performed at 37°C. Cells were then harvesetd into 1 ml tubes, centrifuged at 500 x g for 5 minutes and washed once with 1 ml of PBS to remove unbound reagent. The cells were then resuspended in 500 μl of PBS for analysis on a flow cytometer (Becton Dickinson, LSR Fortessa) with FACS Diva software.

### Terminal deoxyribonucleotidyl transferase (TdT)-mediated dUTP nick-end labelling (TUNEL) assay

TUNEL assay was performed according to the manufacturer’s instructions (ApoAlert^™^ DNA Fragmentation Assay Kit, Takara Bio, Inc., USA). Briefly, 500 μl of KalsomeTM10 treated and untreated promastigotes was harvested by centrifugation and washed twice with 1 ml of PBS. The cells were then resuspended in 0.5 ml of PBS and fixed by adding 5 ml of fresh, prechilled 1% formaldehyde/PBS and incubated at 4°C for 20 min (step repeated two times). Resuspended cells in 0.5 ml of PBS were then permeabilized by adding 5 ml of 70% ice-cold ethanol and incubated at –20°C for at least 4 h. The cells were washed twice in PBS and then transferred to an amber 1.5-ml microcentrifuge tube (to protect samples from light). The cells were then resuspended in 80 μl of equilibration buffer and incubated at room temperature for 5 min. The cells were washed with PBS and resuspended in 50 μl of TdT incubation buffer and incubated at 37°C in a water bath for 60 min, protected from direct light. Then 1 ml of 20 mM EDTA was added to terminate the reaction, and mixed by gentle vortexing. The cells were pelleted down by centrifugation and resuspended in 1 ml of 0.1% Triton X-100/BSA/PBS. The cells were then resuspended gently in 0.5 ml of PI/RNase/ PBS to stain with PI following incubation at room temperature in the dark for 15–30 min. They were analyzed on a flow cytometer, Becton Dickson Canto II with BD FACS Scan Software (San Diego, CA, USA).

### DNA laddering experiment

Fragmentation of DNA into oligonucleosomal bands, as a function of apoptotic cell death, was studied by DNA laddering assay as described before [14]. Apoptotic DNAs from 1 ml of KalsomeTM10 treated and untreated *L*. *donovani* promastigotes (1x10^7^/ml)) were isolated using Suicide Track^™^ DNA Ladder Isolation Kit (Calbiochem, Germany). Briefly, the treated and untreated cells were suspended in 500 μl of extraction buffer which separates apoptotic DNA from high molecular weight DNA. The cells were then incubated on ice for 30 min and centrifuged at 15000 x g for 5 min. Supernatants carefully removed and transferred to a clean tube. RNA in the supernatant was then degraded using solution 2 of the kit. Subsequently, DNA from the cell lysate was isolated using solution 3 and incubation at 50°C for 1 h. The DNA pellet obtained after centrifugation was washed with 70% ethanol followed by 100% ethanol. It was further air dried and resuspended in buffer (10 mM Tris pH 7.5, 1 mM EDTA). DNA aliquots were electrophoresed on 1.5% agarose gel containing ethidium bromide (0.5μg/ml), using Tris—acetate—EDTA (pH 8.0) running buffer, and then observed and photographed under UV illumination in a BioRad gel documentation system (Bio-Rad Laboratories, Inc., Hercules, CA, USA).

### Cell cycle analysis

Cell cycle analysis of KalsomeTM10 treated and untreated *L*.*donovani* promastigotes was done using flow cytometry. After treatment of promastigotes with varying concentrations of KalsomeTM10 for different time periods, the cells were harvested and washed thrice with PBS and fixed in 50% ethanol (diluted in PBS) for 4 h. The fixed cells were then washed thoroughly, treated with 100 μg/ml RNase A and suspended in 1ml of staining solution. After 1 h, the cells were stained with 40 μg/ml PI for 20 min at room temperature. The percentage of cells in G1, S and G2/M phases of the cell cycle were determined in Becton Dickson Canto II flow cytometer and with BD FACS Scan Software.

### Isolation of peritoneal macrophages and in vitro evaluation against intracellular amastigotes

BALB/c mice, 6–8 weeks old were used for the experiments. Mouse peritoneal macrophages were collected by infusing the peritoneal cavity with ice-cold sterile RPMI supplemented with 3% FCS. For in vitro infection studies 5 x 10^6^ peritoneal macrophages were cultured on cover slips and incubated overnight. Next day unattached cells were washed of and then infected with parasites at 1:10 ratio. After 3 h of incubation the unbound parasites were removed by washing thrice with PBS. The infected cells were then treated with increasing concentrations of KalsomeTM10, Ambisome and amphotericin B for 1 h after which the cells were washed with PBS and then incubated in fresh medium for 72 h post infection. The coverslips were then washed twice with PBS and air dried. After methanol fixation the coverslips were stained with giemsa and the infected cells were counted on a light microscope using oil emersion lenses and the images were recorded and processed using Adobe Photoshop 5.5 software.

### Detection of DNA fragmentation in intracellular amastigotes

DNA fragmentation in intracellular amastigotes was analysed by fluorescence microscopy using the In Situ Apotosis Detection Kit (Trevizen, USA). Mouse peritoneal macrophages (1 x 10^6^) were plated in 8-chamber slides (SPL, Korea) and incubated at 37°C in 5% CO2 overnight. Next day unattached cells were washed off and then infected with parasites at 1:20 ratio. After 3 h of incubation the unbound parasites were removed by washing thrice with PBS. The infected cells were then incubated for further 24 h post infection and then treated with KalsomeTM10, Ambisome and amphotericin B for 1h after which the cells were washed with PBS and then incubated in fresh medium for 72 h post infection. The coverslips were then washed twice with PBS and fixed with 3.7% formaldehyde. The slides were then stained using the In Situ Apoptosis Detection Kit and examined using fluorescence microscopy.

### Statistical calculations

All data comparisons were tested for significance using two tailed Student’s t-test using GraphPad software; P values <0.05 were considered significant.

## Results

### Size and size distribution of KalsomeTM10 and Ambisome

Size distribution of liposomal particles is critical to their function [15], therefore, to assess the mean size and size distribution of KalsomeTM10 and Ambisome, NTA measurement with NanoSight LM20 [16] was performed. We found that the mean hydrodynamic diameter of KalsomeTM10 and Ambisome was 119.5± 14.85 nm and 128.0± 1.414, respectively. Moreover, SD of KalsomeTM10 and Ambisome was found to be 56.50± 14.85 and 53.00± 8.485, respectively, indicating that both these liposomal formulations have similar size distribution pattern in addition to their mean size in the range of nm. Numbers representing average values ± standard deviation (n = 2 measurements) (S1 Fig).

### KalsomeTM10 induces killing of *L*. *donovani* promastigotes but not macrophages in a time- and concentration-dependent manner

**Fig 1:**
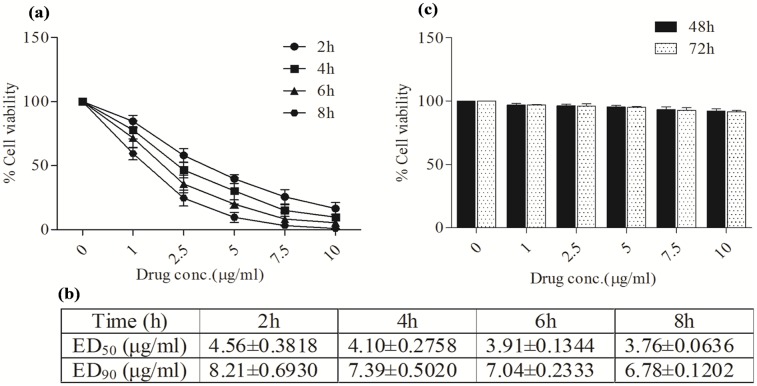
Effect of KalsomeTM10 on the viability of *L*. *donovani* promastigotes and macrophages. (a) Promastigotes were treated with increasing concentrations of KalsomeTM10 at different time points (2, 4, 6 and 8 h) and the percentage cell viability was estimated. (b) The ED_50_ and ED_90_ were determined for each time point. (c) Percent cell viability of murine peritoneal macrophages treated with increasing drug concentrations for 24h and 48h was determined. Results are presented as means±SD; n = 3.

We then investigated the time and dose dependent effect of KalsomeTM10 on promastigotes and macrophages. Treatment of promastigotes for a particular time point with increasing doses of KalsomeTM10 ranging from 0.5–10.0 μg/ml showed a gradual reduction in the cell viability (Fig 1a). Moreover, when the incubation time was increased from 2 to 8 h, drug dose needed for effective killing of the parasite also decreased (Fig 1b). It was found that more than 50% of cells lost viability when treated with 7.5 μg/ml of drug at 2h, which was comparable to those treated with 5.0μg/ml and 2.5 μg/ml of the drug at 4h and 6h, respectively. This suggests that KalsomeTM10 treatment exhibits a time- and concentration- dependent killing of *L*. *donovani* promastigotes. To assess the effect of KalsomeTM10 on mammalian cells, we treated mouse peritoneal macrophages with increasing doses of the drug for 48h and 72h, and measured cell viability. Interestingly, we found no significant killing of macrophages at any of the drug doses used (Fig 1c). Taken together, these results suggest that KalsomeTM10 is efficient in killing *L*. *donovani* promastigotes with no toxic effect on mammalian cells.

### Leishmanicidal effect of KalsomeTM10 in infected macrophages is dose dependent

**Fig 2:**
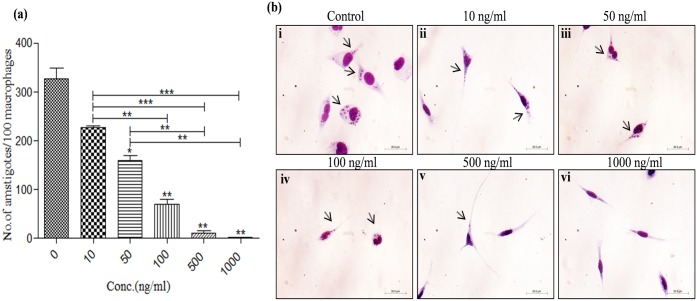
In-vitro leishmanicidal effect of KalsomeTM10 on peritoneal macrophages infected with promastigotes of *L*. *donovani*. Macrophages, infected with promastigotes, were treated with different concentrations of KalsomeTM10 for 72 h post infection. The infected macrophages on coverslips were geimsa stained and counted on a light microscope (a). Number of amastigotes per 100 macrophages was represented as bar graph. Results are presented as means + SD; n = 3. Infected macrophages untreated (b i) and treated with 10 ng/ml (b ii), 50 ng/ml (b iii), 100 ng/ml (b iv), 500 ng/ml (b v) and 1000 ng/ml (b vi) of KalsomeTM10, were giemsa stained and imaged on a light microscope. Pictorial representation of three independent experiments is depicted.

Macrophages are the primary host cells, in which *Leishmania* parasites reside and multiply in the form of amastigotes [17]. Therefore, to assess the efficacy of KalsomeTM10, we treated *L*. *donovani* infected macrophages with increasing doses of the drug and determined the infectivity index (no. of amastigotes/100 macrophages) after 72 h of incubation. We found that KalsomeTM10 exhibits a significant killing of intracellular parasites as revealed by decreasing number of amastigotes in infected macrophages, which was totally cleared of at the highest dose used (Fig 2a). The same was represented as Giemsa stained macrophages where progressive reduction in the no. of intracellular parasites proportionate to the increasing dose of KalsomeTM10 treatment was observed (Fig 2b). The phase contrast micrographs of infected macrophages treated with higher concentrations of KalsomeTM10 has also been shown depicting no much difference in the morphology of the infected macrophages compared to the drug treated infected macrophages (S4 Fig) These results suggest that KalsomeTM10 has potent anti-leishmanial activity against intracellular infection model of VL.

### KalsomeTM10 induces efficient killing of *L*. *donovani* promastigotes and intracellular amastigotes compared to Ambisome and amphotericin B

We then compared the in vitro activity of KalsomeTM10 with Ambisome and amphotericin B, taken as control drugs, against promastigotes and intracellular amastigotes in peritoneal macrophages. Promastigotes and intracellular amastigotes were treated with different doses of these drugs for 4h and 72 h respectively, and ED_50_ and ED_90_ was determined. KalsomeTM10 showed 50 percent (4.73±0.1282 μg/ml, 0.364±0.0140) and 90 percent (7.87±0.2310 μg/ml, 0.6670±0.043) killing of promastigotes and intracellular amastigotes respectively, at a dose of almost half than that required by Ambisome with ED_50_ (7.548±0.1189, 0.862±0.0225) and ED_90_ (13.59±0.2140, 1.552±0.0404), respectively. Although amphotericin B showed 50 percent (0.257±0.0142 μg/ml) and 90 percent (0.437±0.0254) killing of promastigote forms at very low dose, it requires higher concentrations, ED_50_ (2.00±0.0230) and ED_90_ (3.609±0.024), respectively, to exhibit intracellular killing of the parasites compared to KalsomeTM10 with ED_50_ (0.364±0.0140) and ED_90_ (0.6670±0.043), respectively. Moreover, amphotericin B exhibits toxic effect towards macrophages with increasing doses compared to KalsomeTM10 and Ambisome (Table 1).

**Table 1:** KalsomeTM10, Ambisome and amphotericin B differentially affect the viability of *L*. *donovani* parasites and the mammalian macrophages.

Drugs	Promastigotes		Intracellular amastigotes		Toxicity % macrophage survival at 10 μg/ml (±SD)
	ED_50_(μg/ml)	ED_90_(μg/ml)	ED_50_(μg/ml)	ED_90_(μg/ml)	
KalsomeTM10	4.37±0.1282	7.87±0.2310	0.364±0.0140	0.6670±0.043	> 100
Ambisome	7.548±0.1189	13.59±0.2140	0.862±0.0225	1.552±0.0404	> 100
Amphotericin B	0.257±0.0142	0.437±0.0254	2.00±0.0230	3.609±0.024	74.37±3.913

Promastigotes were treated with increasing concentrations of these drugs at 4 h and the percentage cell viability was estimated. The ED_50_ and ED_90_ were determined. Infected macrophages were treated with increasing concentrations of these drugs at 48 h and the no. of parasites per 100 macrophages was determined. The ED_50_ and ED_90_ were determined. Percent cell viability of murine peritoneal macrophages treated with increasing drug concentrations for 24h was determined. Results are presented as means±SD; n = 3.

### Determination of cellular morphology in *L*. *donovani* promastigotes

**Fig 3:**
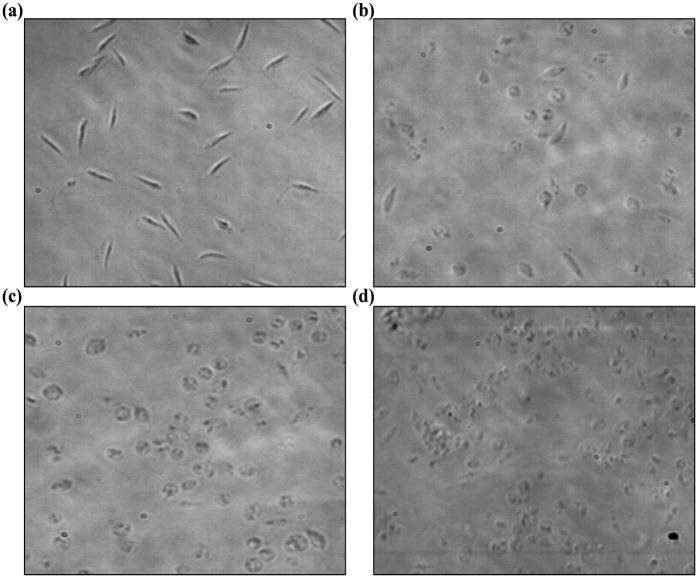
Cellular morphology of KalsomeTM10 treated *L*. *donovani* promastigotes. Phase contrast micrographs of promastigotes after exposure to KalsomeTM10 for 4 h. Untreated control (a), promastigotes exposed to 1.0 μg/ml (b), 2.5 μg/ml (c) and 5.0 μg/ml (d). Images are representative of three independent experiments.

**Fig 4:**
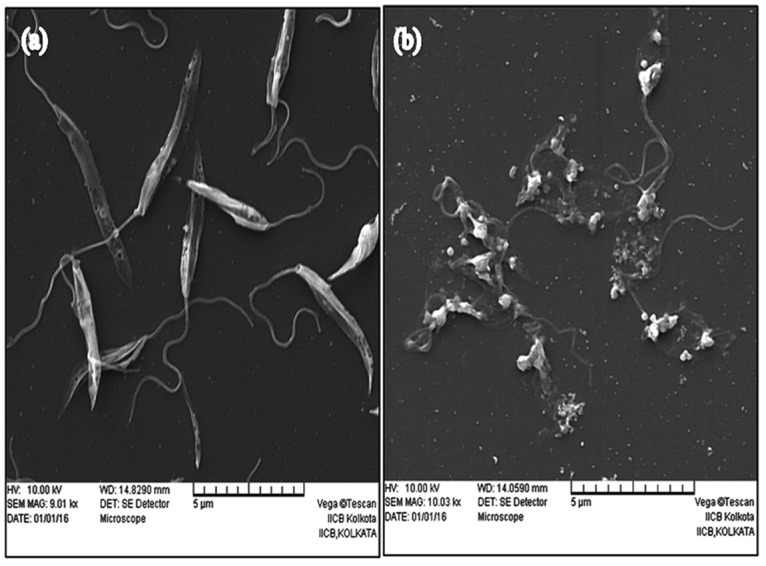
Scanning electron microscopy of promastigotes treated with KalsomeTM10. Promastigotes were either left untreated (a) or treated with 5.0 μg/ml of Kalsome TM10 (b) for 2 h and then analysed for surface topology. SEM micrographs show rounded and distorted shape in KalsomeTM10 treated promastigotes compared to controls. Scale bars: 5 μM. Images are representative of three independent experiments.

We next investigated the mechanism of cell death in KalsomeTM10 treated promastigotes. Morphological changes are one of the key hallmarks that distinguishes apoptotic and necrotic mode of cell death [18]. So, we initially studied the morphological changes in promastigotes treated with different concentrations of KalsomeTM10 at 4 h. Phase contrast micrographs depicted a significant reduction in size and shape of promastigotes along with substantial loss of flagella, at 1.0 μg/ml of drug treatment compared to the untreated controls (Fig 3a and 3b). Promastigotes treated with 2.5μg/ml and 5.0 μg/ml of the drug, displayed complete rounding in shape along with cytoplasmic condensation and shrinkage (Fig 3c and 3d). In addition, SEM analysis also showed a decrease in cell size, loss of flagella, acquisition of ovoid and irregular shape and severe distortion in cell membrane in KalsomeTM10 treated parasites compared to the untreated control, which possessed elongated morphology with anterior flagella and smooth membrane (Fig 4). These observations suggest that KalsomeTM10 induces significant apoptotic-like morphological changes in promastigotes of *L*. *donovani*.

### KalsomeTM10 induces externalisation of membrane-associated phosphatidylserine (PS) in promastigotes of *L*. *donovani*

**Fig 5:**
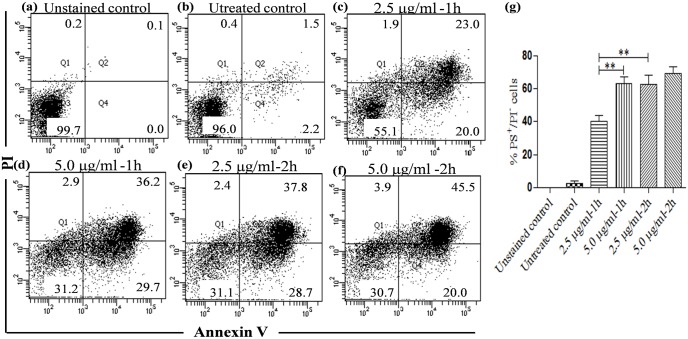
PS externalization in KalsomeTM10 treated promastigotes. Promastigotes, untreated (a and b) and treated with 2.5 μg/ml (c) and 5.0 μg/ml (d) of KalsomeTM10 for 1 h, and treated with 2.5 μg/ml (e) and 5.0 μg/ml (f) of KalsomeTM10 for 2 h, were co stained with annexin V-FITC and PI, and analysed by flow cytometry. The dot plots are representative of one of the three independent experiments. (g) Bar graphs representing mean % PS^+^/PI^−^ cells.**P<0.001.

In the early phase of cellular apoptosis plasma membrane loses asymmetry. This leads to the translocation of phosphatidylserine (PS) from inner to the outer leaflet [19]. Annexin V is a protein which binds to PS. Conversely, PI is a non-permeable dye which selectively enters necrotic cells where it binds to nucleic acids [20]. Therefore, Annexin V-FITC and PI were simultaneously used to assess whether KalsomeTM10 exhibits apoptotic or necrotic mode of cell death in *L*. *donovani* promstigotes. For this, flow cytometry was employed which can distinguish cells undergoing early apoptosis [annexin V (+), PI (-)], late apoptosis [annexin V (+), PI (+)], necrosis [annexin V (-), PI (+)] and those that still remained alive [annexin V(-), PI(-)]. Treatment of promastigotes with KalsomeTM10 at 1 h showed a dose-dependent increase in the percentage of early apoptotic cells [annexin V (+), PI (-)] from 20.0% (2.5 μg/ml) to 29.7% (5.0 μg/ml) compared to the untreated control. However, at 2 h treatment, although early apoptotic cells decreased from 28.7% (2.5 μg/ml) to 20.0% (5.0 μg/ml), a simultaneous increase in the percentage of late apoptotic cells [annexin V (+), PI (+)] from 37.8 (2.5 μg/ml) to 45.5% (5.0 μg/ml) were observed, which indicated that with increase in drug exposure time, cells undergo into the late phase of apoptosis. Interestingly, no significant necrotic cells [annexin V (-), PI (+)] were observed (Fig 5). As a control, when promastigotes were treated with Ambisome (7.5 μg/ml) and amphotericin B (0.25 μg/ml) at 1h it showed 8.7% and 28.3% of early apoptotic cells [annexin V (+), PI (-)], respectively compared to the untreated control (S2 Fig). These results strongly indicate that KalsomeTM10 exhibits an apoptotic-like mode of cell death by inducing externalisation of membrane PS in promastigotes of *L*. *donovani*.

### KalsomeTM10 induces mitochondrial dysfunction by depolarizing mitochondrial membrane potential (Δ*ѱ*_m_) and disrupting mitochondrial integrity with a major impact on depletion of cellular ATP pool in *Leishmania* parasites

**Fig 6:**
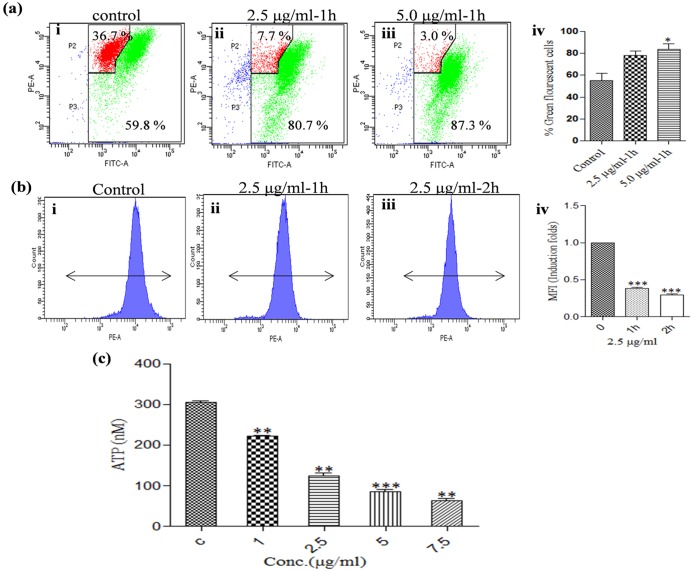
Effect of KalsomeTM10 on mitochondrial membrane potential (Δѱ_m_), mitochondrial integrity and intracellular ATP levels. Promastigotes, untreated (a i) and treated with 2.5 μg/ml (a ii) and 5.0 μg/ml (a iii) of KalsomeTM10 for 1 h, were stained with potentiometric probe JC-1, and analysed by flow cytometry. The dot plots are representative of three independent experiments. Bar graphs representing mean % green flourescent cells (a iv). Promastigotes untreated (b i) and treated with 2.5 μg/ml of KalsomeTM10 for 1 h (b ii) and 2 h (b iii), were stained with mitotraker, and analysed by flow cytometry. Histograms are representative of three independent experiments with fold change depicted as MFI (b iv). Promastigotes were incubated with KalsomeTM10 for 4 h. Cellular ATP concentration was then measured by bioluminescence assay. ATP concentration was expressed as nM (c). Results are presented as means + SD; n = 3. *P<0.05, **P<0.001, ***P<0.0001.

Altered mitochondrial function during apoptosis is linked to a change in the mitochondrial membrane potential [21]. JC-1, a cationic, lipophilic and Δ*ѱ*_m_ sensitive dye, is used to measure such change. In intact mitochondria, JC1, in the form of molecular aggregates, emits red fluorescence, but with depolarisation of mitochondrial membrane potential, it comes out in the cytoplasm, in monomeric form, and emits green fluorescence. This results in the decrease in red/green fluorescence intensity ratio [22]. Thus, to verify the effect of KalsomeTM10 on mitochondrial dysfunction, we monitored mitochondrial membrane potential through flow cytometry. We found that KalsomeTM10 exhibits a dose-dependent decrease in the red/green fluorescence intensity ratio compared to the untreated control at 1h of treatment. This was evident from the percentage of cells in the red region and the green region (36.7% and 59.8% for untreated control, 7.7% and 80.7% when treated with 2.5μg/ml KalsomeTM10 and 3.0% and 87.3% when treated with 5.0 μg/ml of KalsomeTM10, respectively) (Fig 6ai–6aiii). In order to further confirm the involvement of mitochondrial apoptotic pathway in KalsomeTM10 treated promastigotes, we assessed the physical integrity of mitochondria using MitoTracker Deep Red (a stain which enters only actively respiring mitochondria in live cells). Flow cytometry analyses showed that the percentage of Mitotracker positive cells decreased in a time-dependent manner, when promastigotes were treated with 2.5μg/ml of KalsomeTM10 (Fig 6bi–6biii). This could be observed in the form of Mean fluorescence intensity (MFI), whose value fell drastically in the treated cells compared to the untreated control (Fig 6biv). Based on these results, we sought to determine, whether KalsomeTM10 affects the intracellular pool of ATP, as mitochondria are the major contributors to ATP generation, and any disruption in the mitochondrial structure and function would directly affect the intracellular ATP levels [23]. To address this, we treated promastigotes with different doses of KalsomeTM10 at 4 h and measured ATP content using a bioluminiscence kit (Invitrogen). Our results showed that KalsomeTM10 induces a dose-dependent depletion in the total intracellular ATP content, which was to the extent of 27%, 60%, 72% and 80% at 1, 2.5, 5.0 and 7.5 μg/ml of KalsomeTM10 treatment respectively, compared to the untreated control (Fig 6c). Collectively, these results suggest that KalsomeTM10 causes a major mitochondrial dysfunction by inducing depolarisation of mitochondrial membrane potential, loss of mitochondrial integrity and depletion of cellular ATP pool in promastigotes of *L*. *donovani*.

### KalsomeTM10 leads to the generation of intracellular reactive oxygen species (ROS) and lipid peroxidation products in promastigotes of *L*. *donovani*

**Fig 7:**
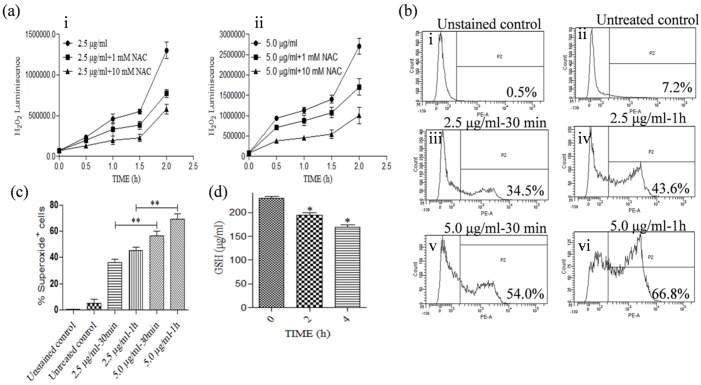
ROS and mitochondrial super-oxide production, and depletion of GSH pool in KalsomeTM10 treated promastigotes. Promastigotes incubated at different time points with KalsomeTM10 at 2.5 μg/ml (a i) and 5.0 μg/ml (a ii), with or without NAC (1 mM and 10 mM). Cellular ROS was measured by bioluminescence assay. Results are presented as means + SD; n = 3. Promastigotes, untreated (b i and ii) and treated with 2.5 μg/ml of KalsomeTM10 for 30 min (b iii) and 1 h (b iv), and treated with 5.0 μg/ml of KalsomeTM10 for 30 min (b v) and 1 h (b vi), were stained with mitosox, and analysed by flow cytometry. The histograms are representative of three independent experiments. (c) Bar graphs representing mean % superoxide^+^ cells. (d) Promastigotes treated with 2.5 μg/ml of KalsomeTM10 at different time points and GSH concentration determined flourometrically. Results are presented as means + SD; n = 3. *P<0.01, **P<0.001.

Oxidoredox homeostasis is essential for cellular survival. During apoptosis altered mitochondrial function results in overproduction of ROS that leads to an oxidative stress and cellular damage [24]. To investigate whether KalsomeTM10 induces ROS generation in leishmanial cells, we measured cellular H_2_O_2_ using a bioluminescence kit (promega). We found that KalsomeTM10 facilitates a dose- (2.5 and 5.0 μg/ml) and time- (0.5 to 2 h) dependent increase in H_2_O_2_ production which however, diminished progressively, when the treatment was carried out in presence of increasing concentrations of the ROS scavenger N-acetyl-L-cysteine (NAC) (1mM and 10 mM) (Fig 7ai and 7aii). We also studied the effect of KalsomeTM10 on mitochondrial superoxide (O_2_^−^) production through flow cytometry. Again, our results showed a dose- and time-dependent increase in mitochondria O_2_^−^ production in leishmanial cells with no such production in untreated control (Fig 7b and 7c). With these results, we sought to verify, whether KalsomeTM10 treatment has any effect on the cellular pool of glutathione (GSH), since redox status in cells is under the tight control of GSH, a potent antioxidant molecule [25]. We measured GSH content in KalsomeTM10 (2.5 μg/ml) treated promastigotes using a flourometric assay kit (Biovision) and found that there was a marked depletion in GSH pool in drug treated parasites compared to the untreated controls (Fig 7d).

**Fig 8:**
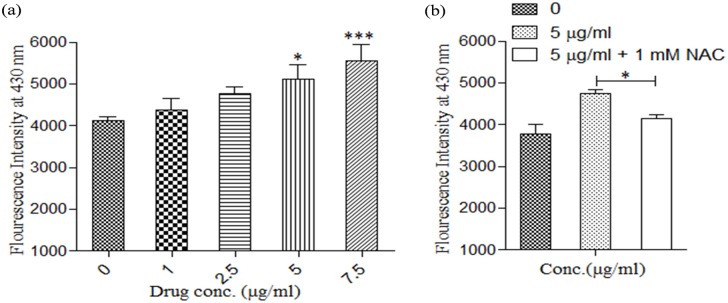
Generation of lipid peroxidation products on treatment with KalsomeTM10. Promastigotes were treated with different concentrations of kalsomeTM10 for 2 h in the absence (a) and presence of NAC (b), and lipid peroxidation products were determined flourometrically. Results are presented as means + SD; n = 3. *P*<0.05.

Lipid peroxidation, a process in which oxidants such as free radicals target polyunsaturated fatty acids resulting in lipid peroxyl radicals and hydroperoxides generation, is detrimental to cells leading to their death either through apoptotic or necrotic mode [26]. Thus, to assess whether KalsomeTM10 induces generation of lipid peroxidation products, we treated promastigotes at 2 h with varying doses of the drug and measured lipid peroxidation products flourometrically. Our results showed that KalsomeTM10 induces a dose-dependent increase in these products (Fig 8a). To further confirm that KalsomeTM10 does so by generating ROS, we treated promastigotes with KalsomeTM10 in the presence of NAC. The results showed diminished production of lipid peroxidation products in the presence of NAC, indicating that KalsomeTM10 exhibits potent ROS generation in promastigotes that leads to the production of these products (Fig 8b). Altogether, these observations suggest that KalsomeTM10 mediated mitochondrial dysfunction results in over production of free radicals with concomitant depletion of intracellular GHS content and increased generation of lipid peroxidation products, which triggers apoptotic-like mode of cell death in promastigotes.

### Induction of in vivo caspase-like activity in *L*. *donovani* by KalsomeTM10

**Fig 9:**
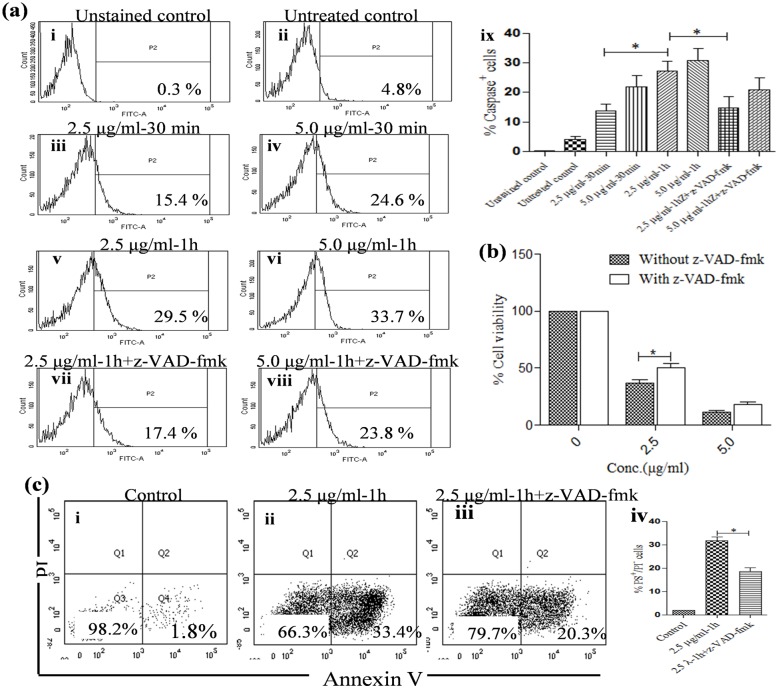
Evaluation of caspase activity in KalsomeTM10 treated *L*. *donovani* promastigotes. Promastigotes were either left untreated (a i and ii) or treated with 2.5 μg/ml (a iii) and 5.0 μg/ml (a iv) of kalsomeTM10 for 30 min and treated for 1h in absence or presence of z-VAD-fmk with 2.5 μg/ml (a v and vi) and 5.0 μg/ml (a vii and viii) of kalsomeTM10, respectively. Caspase positive cells were analysed through flow cytometry and plotted as histograms which are representative of three independent experiments. (a ix) Mean caspase positive cells represented as bar graphs. (b) Percent cell viability was determined in promastigotes treated with different concentrations of KalsomeTM10 for 2 h in the absence and presence of z-VAD-fmk. Results are presented as means + SD; n = 3. Promastigotes, either untreated (c i) or treated with 2.5 μg/ml kalsomeTM10 for 1 h in the absence (c ii) and presence of z-VAD-fmk (c iii), were co stained with annexin V-FITC and PI, and analysed by flow cytometry. The dot plots are representative of three independent experiments. (c iv) Mean PS^+^/PI^−^ cells represented as bar graphs. *P<0.01.

Caspases are one of the key molecules which orchestrate the process of apoptosis [27]. Thus to determine, if caspases really play any role in KalsomeTM10 mediated cell death in promastigotes, we used Apostat (a cell permeable caspase specific substrate with flourescent tag; from R&D), which detects intracellular active caspase. Flow cytometric analyses showed that KalsomeTM10 treatment induces a dose- and time- dependent increase in caspase positive cells, which was significantly reduced by the pan caspase inhibitor, z-VAD-fmk, suggesting that KalsomeTM10 activates caspase mediated apoptotic machinery in promastigotes of *L*. *donovani* (Fig 9a). To further confirm the involvement of caspases, we performed cell viability assay in the presence and absence of z-VAD-fmk. Our results showed that z-VAD-fmk significantly reduced cell death in promastigotes at 1h treatment of KalsomeTM10 (2.5 μg/ml) (Fig 9b), which also reflected in the decrease in the percentage of annexin-v positive cells (Fig 9c). Treatment of promastigotes with Ambisome (7.5 μg/ml) and amphotericin B (0.25 μg/ml) at 1h also showed induction of caspase positive cells (S3 Fig). These results suggest that KalsomeTM10 mediated cell death in promstigotes of *L*. *donovani* is caspase dependent.

### Determination of apoptotic-like cell death by oligonucleosomal DNA fragmentation in *L*. *donovani* promastigotes

**Fig 10:**
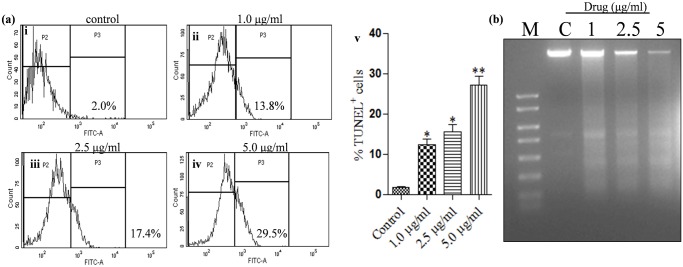
Fragmentation of genomic DNA in KalsomeTM10 treated promastigotes analysed through TUNEL assay and DNA laddering assay. Promastigotes were either left untreated (a i) or treated with 1.0 μg/ml (a ii) 2.5 μg/ml (a iii) and 5.0 μg/ml (a iv) of KalsomeTM10 for 12 h, and then TUNEL assay was performed. The fluorescence was analysed by flow cytometry and presented as histograms which are representative of two independent experiments. (b) Mean TUNEL+ cells represented as bar graphs. (c) Promastigotes treated with different concentrations of KalsomeTM10 for 14 h. Apoptotic DNA was resolved on a 1.5% agarose gel. Representative images of three independent experiments. *P<0.01, **P<0.001.

The cleavage pattern of genomic DNA characterized by fragmentation into nucleosomal units is a distinctive feature of apoptotic cell death [28]. To investigate whether KalsomeTM10 exhibits DNA fragmentation in *L*. *donovani* promastigotes, we performed TUNEL staining that detects fragmented DNAs by labelling their free ends with dUTP. We found that KalsomeTM10 significantly increased TUNEL positive cells in a dose-dependent manner compared to the untreated control cells (Fig 10a). To further substantiate this observation, we also performed DNA laddering assay, by employing agarose gel electrophoresis. The results showed that there was marked fragmentation in genomic DNA into oligonucleosome-sized fragments (in multiples of 200 bp) when promastigotes were treated with 1.0 μg/ml (lane 3), 2.5 μg/ml (lane 4), and 5.0 μg/ml (lane 5) of KalsomeTM10. In contrast, untreated cells (lane 2) did not show any DNA fragmentation (Fig 10b). Moreover, it is evident from Fig 10b that DNA laddering initiated at the lowest drug dose and with further increase in the dose, there was a marked decrease in the size of the intact DNA with a corresponding increase in the DNA laddering when compared to the untreated controls. Overall, these results suggest that KalsomeTM10 causes genomic DNA fragmentation, which might contribute to the induction of apoptotic-like cell death in *L*. *donovani* promastigotes.

### KalsomeTM10 induces cell cycle arrest in *L*. *donovani* promastigotes

**Fig 11:**
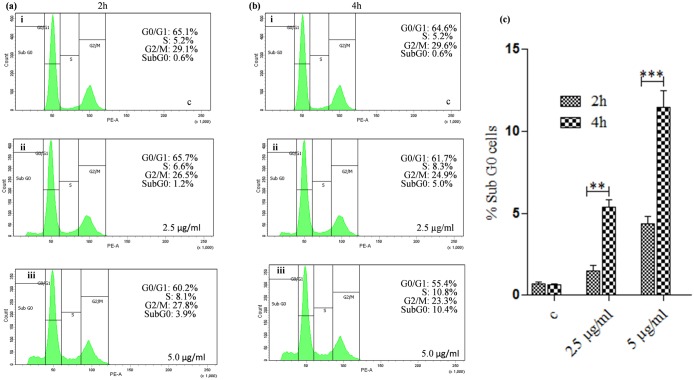
Analysis of cell cycle arrest in *L*. *donovani* promastigotes treated with KalsomeTM10. Promastigotes, either left untreated (a i) or treated with 2.5 μg/ml (a ii) and 5.0 μg/ml (a iii) of kalsomeTM10 for 2 h and untreated (b i) or treated with 2.5 μg/ml (b ii) and 5.0 μg/ml (b iii) of KalsomeTM10 for 4 h, were analysed for cell cycle through flow cytometry and plotted as histograms which are representative of three independent experiments. (c) Mean % Sub G0 cells represented as bar graphs.**P<0.001, ***P<0.0001.

Degradation of genomic DNA has profound effects on the replication of cells [29]. To verify whether KalsomeTM10 affects promastigote’s replication, we treated parasites with different doses of the drug at varying time points, and analysed cell cycle progression through flow cytometry. The results indicated that KalsomeTM10 induces a dose- and time-dependent arrest of leishmanial cells, since the percentage of cells in the sub-G0 phase of the cell cycle increased when compared to the untreated control (Fig 11a and 11b).

### KalsomeTM10 induces apoptotic DNA fragmentation in intracellular amastigotes of *L*. *donovani*

**Fig 12:**
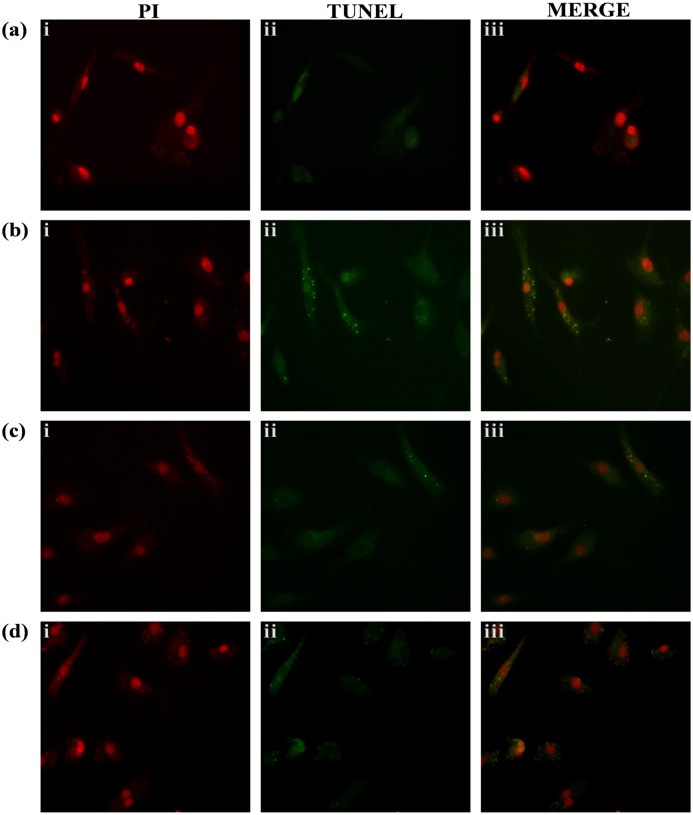
Analysis of genomic DNA fragmentation in KalsomeTM10, Ambisome and amphotericin B treated intracellular amastigotes analysed through TUNEL assay. *L*. *donovani* infected murine peritoneal macrophages were either left untreated (a) or treated with KalsomeTM10 (b) at 0.5 μg/ml, Ambisome (c) at 1.0 μg/ml and amphotericin B (d) at 2.0 μg/ml. After 48 h, infected macrophages were stained using TUNEL and analysed by fluorescence microscopy. Total nuclei are visualized in red (PI) and TUNEL-positive nuclei are stained in green (FITC). The images are representative of three independent experiments.

To determine the mechanism of killing of intracellular amastigotes, peritoneal macrophages were infected with stationay phase promastigotes of *L*. *donovani*. After 24h of incubation, infected macrophages were treated with KalsomeTM10 and control drugs Ambisome and amphotericin B for 48h. TUNEL assay show that compared to untreated infected macrophages (Fig 12a) KalsomeTM10 treatment induces DNA degradation into apoptotic fragments as revealed by prominent green signal in the amastigotes without affecting macrophage DNA (Fig 12b). Ambisome (Fig 12c) and amphotericin B (Fig 12d) also induced DNA fragmentation but with low efficacy compared to KalsomeTM10 as revealed by the moderate green signal intensity.

### KalsomeTM10 mediated killing of intracellular amastigotes is ROS and nitric oxide dependent

**Fig 13:**
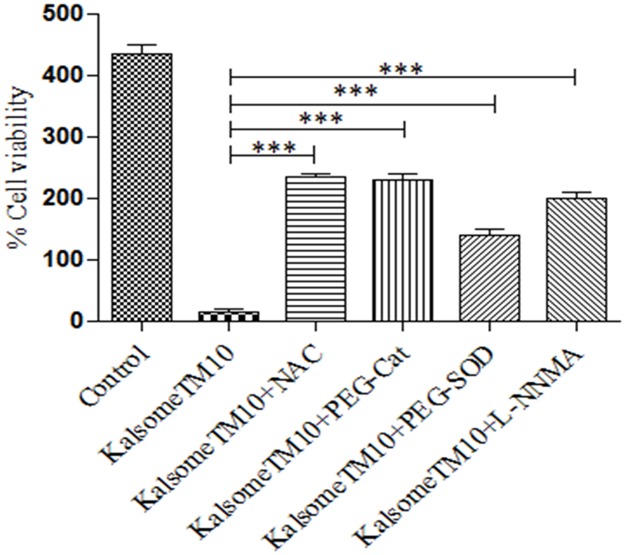
Role of ROS and nitric oxide generation on in-vitro leishmanicidal effect of KalsomeTM10 on intracelluar amsastigotes. Macrophages, infected with promastigotes, for 24 h were pretreated with ROS scavangers like NAC (10 mM), PEG-Cat (500 units) and PEG-SOD (500 units) and nitric oxide inhibitor L-NMMA (2 mM) for 30 mins and then treated with KalsomeTM10 (0.5 μg/ml) for 1h. After washing, incubation for 48 h post drug treatment agan in the prescence of these scavangers and inhibitor was carried out and then the infected macrophages were geimsa stained and counted on a light microscope to determine infectivity index (Number of amastigotes per 100 macrophages) that was represented as bar graph. Results are presented as means + SD; n = 2.***P<0.0001.

ROS and nitric oxide are potent anti-leishmanial molecules [30,31]. Therefore, to verify if kalsomeTM10 induces generation of these molecules as a probable mechanism of killing intracellular amastigotes, peritoneal macrophages were infected with promastigotes of *L*. *donovani*. After 24 h of incubation, infected macrophages were treated with KalsomeTM10 in absence and presence of the ROS scavangers (NAC, PEG-Cat and PEG-SOD) and nitric oxide synthesis inhibitor (L-NMMA) for 48 h and the infectivity index (no. of amastigotes/100 macrophages) was determined. We found that KalsomeTM10 mediated killing of intracellular parasites was significantly inhibited in the presence of the above mentioned ROS scavengers and nitric oxide synthesis inhibitor revealing that KalsomeTM10 employs these antimicrobial molecules to kill the parasites (Fig 13).

### Endocytosis play important role in KalsomeTM10 mediated but not amphotericin B mediated killing of intracellular amastigotes

**Fig 14:**
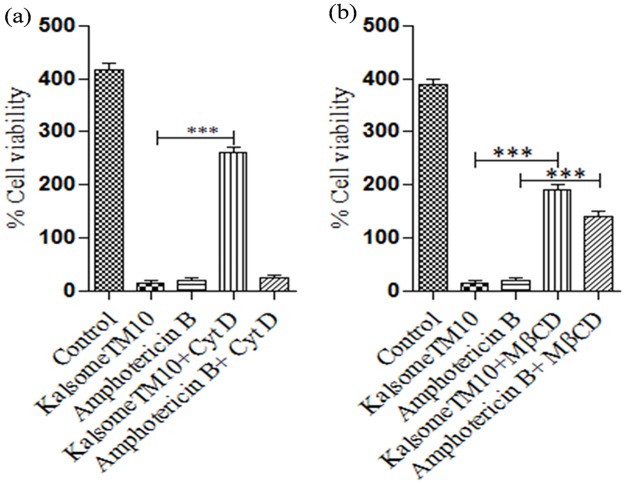
Effect of cytochalasin D and MβCD on in-vitro leishmanicidal effect of KalsomeTM10 and amphotericin B on intracelluar amsastigotes. Macrophages, infected with promastigotes, for 24 h were pretreated with cytochalasin D (25 mM) (a) and MβCD (20 mM) (b) for 30 mins and then treated with KalsomeTM10 (0.5 μg/ml) and amphotericin B (3 μg/ml) for 1h in cytochalasin D treted macrophage and for 30 mins in MβCD treated macrophages. After washing, incubation for 48 h post drug treatment in the prescence of these scavangers and inhibitor was carried out and then the infected macrophages were geimsa stained and counted on a light microscope to determine infectivity index (Number of amastigotes per 100 macrophages) that was represented as bar graph. Results are presented as means + SD; n = 2. ***P<0.0001.

Endocytosis of liposomal formulations to cells is crucial to their function [32,33]. Therefore, to evaluate if endocytosis is necessary for kalsomeTM10 and amphotericin B mediated anti-leishmanial effects, peritoneal macrophages were infected with promastigotes of *L*. *donovani*. After 24 h of incubation, infected macrophages were pretreated with cytochalasin D for 1h and then treated with KalsomeTM10 and amphotericin B for 1h. After washing the cells, further incubation for 48 h was carried out and the infectivity index (no. of amastigotes/100 macrophages) was determined. We found that KalsomeTM10 mediated killing of intracellular parasites was significantly inhibited in the presence of the cytochalasin D but no such inhibition was observed in amphotericn B treated macrophages, indicating that endocytosis has crucial role in KalsomeTM10 mediated anti-leishmanial effect (Fig 14a).

### Cholesterol depletion on host macrophage diminished KalsomeTM10 and amphotericin B mediated killing of intracellular amastigotes

Amphotericin B binds to membrane cholesterol [32], therefore, to determine if cholesterol depletion of macrophage affects the leishmanicidal function of kalsomeTM10 and amphotericin B, peritoneal macrophages were infected with promastigotes of *L*. *donovani*. After 24 h of incubation, infected macrophages were pretreated with MβCD for 40 mins and then treated with KalsomeTM10 and amphotericin B for 1h. After washing the cells, further incubation for 48 h was carried out and the infectivity index (no. of amastigotes/100 macrophages) was determined. We found that not only amphotericn B but also KalsomeTM10 mediated killing of intracellular parasites was significantly inhibited in cholesterol depleted macrophages, indicating that membrane cholesterol plays crucial role in KalsomeTM10 and amphotericn B mediated anti-leishmanial effect (Fig 14b).

## Discussion

The in vivo anti-leishmainal efficacy of KalsomeTM10 that we reported in our previous work, prompted us to investigate the relevant cell death mechanism, by which KalsomeTM10 induces killing of the parasites without affecting the host. To our knowledge no such study using a liposomal formulation of amphotericin B has been reported that show induction of apoptotic-like cell death in both promastigotes as well as intracellular amastigotes of *L*. *donovani*. Though, induction of programmed cell death in promastigotes of *L*. *donovani* in response to amphotericin B treatment only, has been described [34], but again, its mechanism of killing on the intracellular amastigotes is lacking. Even Ambisome, (another liposomal amphotericin B), which we took as a control, and that was not previously shown, was found to induce similar kind of apoptotic changes in both promastigotes as well as intracellular amastigotes as indued by KalsomeTM10. However, for attaining similar kind of anti-leishmanial response, concentration of Ambisome required was almost double than that of KalsomeTM10. This indicates to the potential of KalsomeTM10 over Ambisome, since KalsomeTM10 can meet therapeutic dose of amphotericin B without increasing the amount of lipids (used for the delivery of the free drug), thereby; lowering the risk of lipid associated side effects on the host [35,36].

The mechanistic aspect of cell death in KalsomeTM10 treated promastigotes depicted apoptotic like morphological alterations similar to several anti-leishmanials [37,38] with reduction in the body length, rounding of cells, loss of flagella and cell shrinkage etc. Further, study on PS externalisation, an early event of apoptosis [39,40] showed that KalsomeTM10 can exhibit this in a dose- and time-dependent manner. Interestingly, no PI positive cells were observed indicative of an apoptotic-like mode of cell death. Ambisome treatment also showed PS exposure. However, the efficacy of Ambisome was significantly lower compared to KalsomeTM10 and amphotericin B. This was evidenced by the percentage of PS positive cells (8.7%) observed after Ambisome treatment at 7.5 μg/ml, which was ~3 fold less than the percentage of PS positive cells after kalsomeTM10 (28.7%) and amphotericin B (28.3%) treatment at 5 μg/ml and 0.25 μg/ml, respectively. One plausible mechanism for this could be the delayed accessibility of free amphotericin B to the *Leishmania* membrane [32], as amphotericin B alone at a very low dose of 0.25 μg/ml manifested comparable amount of PS positive cells, to that shown by 5 μg/ml amphotericin B when encapsulated in KalsomeTM10. Remarkable differences between the action of Ambisome and KalsomeTM10 may be explained by the lamelarity as well as cholesterol content of these two liposomal formulations. Ambisome is a unilamelar bilayer liposome containing cholesterol, where amphotericin B is intercalated within the membrane bilayer [11]. However, KalsomeTM10 is a multilamalar liposome [8], where in addition to the higher amount of intercalated amphotericin B due to ergosterol, a significant amount of soluble amphotericn B can be present in the aqueous compartment between the lipid bilayers making the possibility of membrane accessible amphotericn B significantly higher [32]. Despite these results showing varying degree of PS exposure on promastigotes induced by KalsomeTM10, Ambisome and Amphotericin B treatment, we cannot exclude the prescence of other phospholipids including phosphatidylglycerol and phosphatidyl inositol-4, 5 bisphosphate. This is because Annexin V can bind to these lipids also [41]. Presence of PS on the membrane of *L*. *donovani* is still a matter of debate. Though Weingartner showed absence of PS [42], Imbert et al. showed that in addition to other phospholipids PS is also present in the membrane of *L*. *donovani* promastigotes [43]. Interestingly, the study carried out by Banerjee et al. showed that PC-SA liposome having high affinity towards PS (confirmed by incubating with PS and other phospholipid liposomes) failed to significantly kill promastigotes preincubated with annexin V [44]. This indirectly shows that in comparison to other phospholipids, PS is in fact present in significant amount on the surface of promastigote where annexin V majorly binds in addition to other lipid moieties. Presence of PS in promastigotes of *L*. *major* was depicted through the use of annexin V, PS-specific antibody and protein S [45]. Thus, our results using annexin V binding assay suggests that treatment of promastigotes with KalsomeTM10, Ambisome and amphotericin B, lead to higher exposure of the annexin V binding phospholipids (majorly PS) on the surface of the parasites thus causing apoptotic-like cell death in these parasites. Similar to this, recently, many studies have shown PS exposure in dying parasites through annexin V binding assay upon treatment with different anti-leishmanials [46–48].

Mitochondria play crucial role in cell survival and death [49]. Since *Leishmania* possess a single, large mitochondrion that responds to majority of their (ATP) energy requirements, it represents a potential drug target [50]. We showed that KalsomeTM10 can induce a remarkable decrease in mitochondrial membrane potential as well as disruption in mitochondrial physical and structural integrity. Permeabilisation of mitochondrial outer membrane is critical to the mitochondrial signaling leading to cell death [24], and since lipid vehicles bear fusogenic property with the hydrophobic membranes [15], hence forth, it can be possible that the mitochondrial outer membrane would be highly sensitive to KalsomeTM10. This can be reflected by the drug exposure time that within 1 h of KalsomeTM10 treatment, a drastic fall in the red fluorescence (JC1 aggregates), indicating to the loss of mitochondrial membrane potential was observed [51]. But how KalsomeTM10 is internalised into the promastigotes is still not known which requires additional study. Further, the observation that KaslomeTM10 treatment exhibited ATP depletion could be the result of the disruption in the mitochondrial transmembrane potential (the electrochemical gradient that drives ATP synthesis) [18]. Altogether, these indicate that a loss in mitochondrial membrane potential has a significant impact on leishmanial membrane integrity [49,50,52] probably by decondensation of the kinetoplasts which are held by the inner-membrane [53], and thus hampering ATP generation. LQB 118 treated promastigotes of *L*. *amazonensis* reported similar dissipation of mitochondrial membrane potential causing cellular ATP depletion [52]. Uncoupling of mitochondrial oxidative phosphorylation is a major contributing factor to the generation of free radicals [54]. We also found that KalsomeTM10 induces a rapid increase in H_2_O_2_ as well as superoxide production in a dose and time dependent manner. This was further confirmed by the use of NAC (ROS quencher), which significantly reduced H_2_O_2_ production. These results are in accordance with Ribero et al [52], who previously demonstrated that LQB-118 induces increased generation of ROS in *L*. *amazonensis* during the early hours of treatment to trigger apoptosis. Further, evaluation of antioxidants like GSH, which protects kinetoplastids by buffereing excessive ROS concentration [55,56], showed that KalsomeTM10 significantly depleted GSH content in promastigotes. In the absence of antioxidants, ROS tends to impart detrimental effects on the integrity of various lipid molecules. Lipid peroxidation products thus generated, further fuel the cell death mechanism [26]. KalsomeTM10 displayed enhanced generation of these products which diminished in the presence of NAC. Overall, these suggest that KalsomeTM10 employs mitochondrial dependent pathway with major impact on intracellular ATP pool to exhibit apoptotic-like cell death in promastigotes of *L*. *donovani*.

Caspases, a specific family of cysteine proteases, play important role in executing mammalian apoptosis [57]. However, their implication in *Leishmania* apoptosis is controversial. Although some investigators demonstrated caspase-like activity others have reported caspase independent apoptotic cell death in *Leishmania* promastigotes [34,58,59]. We showed using ApoStat, a pan caspase inhibitor earlier employed to detect mammalian cell apoptosis [60,61], that KalsomeTM10 can induce a dose- and time-dependent increase in the percentage of caspase positive cells, which decreased significantly in the presence of the broad caspase inhibitor zVAD-fmk. Ambisome treatment for 1h also showed caspase induction (16.0% cells) at 7.5 μg/ml which was comparable to that induced by amphotericin B (16.4%) at 0.25 μg/ml. KalsomeTM10 however depicted higher percentage of caspase positive cells (29.5%) at 2.5 μg/ml, compared to Ambisome. Similar in vivo capase-like activity, in terms of PPL cleavage, in amphotericin B treated promastigotes has been documented [34]. Therefore, all these observations indicate that though the genome of *Leishmania* does not encode classical caspases [62], existence of proteases similar to the mammalian caspases, where this inhibitor binds, could not be excluded. Like caspases, metacaspases have been reported in protozoan parasites [63,64] but their role in *Leishmania* apoptosis is not clear. So it is unlikely to infer that ApoStat binds to these enzymes. Rather, possibility towards other putative proteases could exist, since this inhibitor can bind non-specifically to proteases like calpain, cathepsin B and cathepsin H etc. [65,66]. Nevertheless, to further confirm the involvement of caspase-like activity in KalsomeTM10 mediated killing of promastigotes, the parasites were incubated with the z-VAD-fmk during drug treatment. z-VAD-fmk significantly downregulated killing of the promastigotes as well as PS exposure. Despite this, the role of z-VAD-fmk in inhibiting other proteases like cathepsin B [66] cannot be excluded. Altogether it reveals that KalsomeTM10 induces apoptotic-like cell death in promastigotes of *L*. *donovani* in part via caspase dependent pathway.

Apoptotic DNA fragmentation in KalsomeTM10 treated promastigotes analysed through TUNEL assay [67] showed progressive increase in the percentage of tunel positive cells. This was further confirmed through DNA laddering assay which showed oligonuleosomal bands of fragmented DNAs. Progressive DNA laddering profile was clearly visible in cells treated with increasing drug concentrations while such patterns were not visible in the control profile. We further showed that KalsomeTM10 can induce cell cycle arrest which is likely a result of genomic DNA fragmentation that inhibits cell proliferation [68]. Flow cytometry analysis revealed that the promastigotes enter into the sub Go stage and get arrested therein, without really entering into the mitotic (M) phase with an increase in the dose as well as time of incubation. This could be due to the activation of different nucleases which in turn facilitate oligonuleosomal fragmentation of genomic DNA, and effectively block DNA replication during the S phase of the cell cycle [68]. Taken together, DNA fragmentation and cell population arrest at sub Go/G1 phase of cell cycle, both hallmarks of classic apoptosis, confirm the apoptotic-like cell death in *L*. *donovani* promastigotes upon KalsomeTM10 treatment.

The various biochemical markers confirming mechanism of cell death in promastigotes led us to analyze the mechanism of cell death in intracellular amastigotes. Interestingly, we found that KalsomeTM10 induces degradation of parasite’s genomic DNA into apoptotic fragments as determined by Insitu TUNEL assay, without any effect on host cell DNA. The DNA fragmentation was more prominent compared to Ambisome and amphotericin B treated amastigotes. One previous study has shown *L*. *donovani* metacaspase-1 to be an effector molecule in apoptosis like cell death in the parasite [69]. We also found that KalsomeTM10 treatment of *L*. *donovani* infected macrophages induces metacaspase-1 expression in the intracellular parasites which was ~ 16.5 folds higher compared to the untreated control (S5 Fig) Taken together, these suggest that KalsomeTM10 also induces apoptotic-like cell death in the intracellular amastigotes. Investigation of probable mechanism for this showed that KalsomeTM10 mediated clearance of intracellular amastigotes was ROS and nitric oxide dependent, as the presence of specific scavangers and inhibitors of ROS and nitric oxide synthesis respectively, significantly inhibited KalsomeTM10 induced killing of the parasites. However, whether amastigotes-KalsomeTM10 interaction is necessary for the anti-leishmanial effect is not clear. Therefore, to further delineate whether endocytosis is indispensable in KalsomeTM10 mediated killing of the intracellular amastigotes, cytochalasin D was used. Intriguingly, KalsomeTM10 induced killing of these parasites was significantly inhibited by cytochalasin D indicating endocytosis to be important for its anti-leishmanial effect in host macrophage. However, no such inhibition upon amphotericn B treatment was observed. But how endocytosis of KalsomeTM10 is taking place is not yet clear. Moreover, any receptor present on macrophage cell surface which might be involved in uptake of this liposomal formulation after binding to some of its specific component is still not known. Previous studies on uptake of other liposome based formulations like polyethylene glycol modified liposome encapsulating hemoglobin has shown to undergo scavenger receptor mediated endocytosis in monocytes and macrophage cells [15,70]. So, it can be speculated that some surface receptor of host cell might be involved in endocytosis of KalsomeTM10. Furthermore, lack of complete inhibition in killing of parasites by cytochalasin D may be because of the pinocytosis [15] of the smaller sized particles present in KalsomeTM10 formulation. Nevertheless, this show that inhibition of endocytosis prevented entry of optimum amount of KalsomeTM10 into the cell (as most of them were washed off after 1h treatment) resulting in no significant killing of the parasites. However, binding of amphotericn B to the membrane cholesterol was strong enough to withstand its dispersal during washing. Therefore, once bound it may enter into the cells at later time point through endocytosis [33] to exhibit its anti-leishmanial effect. To further strengthen this observation, we then evaluated the effect of depletion of the macrophage membrane cholesterol (using MβCD) on the killing activity of KalsomeTM10 and amphotericin B, with the notion that it would prevent amphotericn B binding to the membrane. Surprisingly, cholesterol depletion not only inhibited amphotericin B mediated but also KalsomeTM10 mediated killing of the parasites significantly. But how this is happening is not still known. It can be speculated that due to the distortion of receptors (in cholesterol depleted membrane) [71], the liposomal components may not bind to these receptors. However, if they do bind somehow, they might not be internalized due to the aberrant recruitment of the receptors in the disrupted lipid rafts [72].

Overall this study show that inspite of showing prompt killing of *Leishmania* promastigotes, amphotericin B has cytotoxic effects on mammalian cells at the therapeutic dose. However, the liposomal amphotericin B showed difference in its sensitivity towards these two kinds of cells, wherein it induced apoptosis in *leishmanial* cells but not in the mammalian macrophages. Hence, it depicts that the delivery systems like liposomes modify the effect of amphotericin B depending on the cell types. The reason for this difference is not clear. Fusion of lipid vesicles allowing easy accessibility of free amphotericin B [32] to the *Leishmania* membrane may explain this observation. Nevertheless, this study shed new light into the previous perception of using lipid vesicles just as a delivery system for anti-leishmanial drugs like amphotericin B, at least when mechanism of killing of promastigotes of *Leishmania* is considered. Moreover, since the accessibility of these lipid based vehicles to the intracellular amastigotes cannot be excluded [73], understanding their interaction with the promastigotes form could provide new insight into the killing mechanism of the intracellular parasites. Future study on the macrophagic modulations at the mechanistic level would allow better understanding of the in vivo antimicrobial functions of KalsomeTM10. Moreover, investigating the potency of KalsomeTM10 further on promastigotes of other *Leishmania* species like *L*. *amazonensis*, *L*. *major*, *L*. *brazillensis*, etc would widen the scope of this novel liposomal amphotericin B and could open up avenues for the development of similar new anti-leishmanials.

## Supporting information

S1 Methods Supplementary methods. (DOCX)

S1 Fig Size distribution from NTA measurements of the two liposomal formulations. KalsomeTM10 (A-C) and Ambisome (D-F) (left panels) with the corresponding NTA video frame (right panels) and 3D graph (size vs. intensity vs. concentration; (middle panels).
(TIF)

S2 Fig PS externalization in Ambisome and amphotericin B treated promastigotes. Promastigotes, untreated (a and b) and treated with Ambisome at 7.5 μg/ml (c) and amphotericin B at 0.25 μg/ml (d) for 1 h, were co stained with annexin V-FITC and PI, and analysed by flow cytometry. The dot plots are representative of two independent experiments (e) Bar graphs representing mean % PS^+^/PI^−^ cells. **P<0.001, ***P<0.0001.
(TIF)

S3 Fig Evaluation of caspase activity in Ambisome and amphotericin B treated *L*. *donovani* promastigotes. Promastigotes were either left untreated (a and b) or treated with Ambisome at 7.5 μg/ml (c) and amphotericin B at 0.25 μg/ml (d) for 1 h. Caspase positive cells were analysed through flow cytometry and plotted as histograms which are representative of two independent experiments. (e) Bar graphs representing mean % caspase^+^ cells. ***P<0.0001.
(TIF)

S4 Fig Phase contrast micrographs of peritoneal macrophages infected with promastigotes of *L*. *donovani*. Macrophages, infected with promastigotes, were either untreated (A) or treated with 500 ng/ml (B) and 1000 ng/ml of KalsomeTM10 for 72 h post infection. The images of these cells were captured under a light microscope.
(TIF)

S5 Fig Determination of metacaspase-1 gene expression in intracellular amastigotes upon KalsomeTM10 treatment. RAW 264.7 macrophages infected with promastigotes of *L*. *donovani* were either untreated or treated with 500 ng/ml of KalsomeTM10 for 2 h. The change in gene expression was determined through Real-time PCR using specific primers for *L*. *donovani* metacaspase-1and GAPDH (used as internal control). **P<0.05.
(TIF)
