# Supplementary material for: Apoptosis-like cell death in Leishmania donovani treated with KalsomeTM10, a new liposomal amphotericin B
Source: PLoS One. 2017 Feb 7;12(2):e0171306. doi: 10.1371/journal.pone.0171306 (PMC5295687; doi:10.1371/journal.pone.0171306)
Supplement: S1 Methods — (DOCX) [file pone.0171306.s001.docx]

**Materials and methods**

**Culture of RAW 264 macrophage cell line and drug treatment of *L. donovani* infected macrophage**

The murine macrophage like cell line RAW 264.7 was cultured in 90 mm culture dishes in 10-15 ml RPMI medium containing 10% FBS. Using cell scraper the cells were detached and counted. A total of 10^5^ cells in 500 µl medium per well of a 24 well culture plate was plated overnight. Next day, the cells were infected with *L. donovani* promastigotes at 1:10 ratio. After 24 h of infection the infected cells were either left untreated or treated with 500 ng/ml of KalsomeTM10 for 1h. After washing the cells with medium, incubation for additional 1h was done. The cells per two wells were then lysed in 500 µl Trizol reagent and stored in -20 ^0^C till RNA isolation.

**Isolation of total RNA, cDNA synthesis and Real-time PCR**

Total RNA from cells (untreated or drug treated) were isolated using Trizol reagent (Invitrogen), according to the manufacturer’s instructions. A total of 2 µg of RNA from each sample was reverse transcribed to cDNA using the iScript cDNA synthesis kit (Bio-rad), according to the manufacturer’s protocol. Using LightCycler 96 (Roche), Real-time PCR was performed according to the SYBER GREEN method (KAPA BIOSYSTEMS). The PCR thermocycling parameters were kept as 95°C for 10 min, 45 cycles of 95°C for 15 s, 50°C for 30 sec and 72°C for 10 sec. GAPDH was used as internal control. Samples were run in duplicates. The fold induction was determined by 2^- ∆∆CT^ method (Thomas et al, 2008). Fold induction of duplicate samples were averaged.

Primer sequences for *L. donovani* specific genes are:

GAPDH (glyceraldehyde-3-phosphate dehydrogenase): forward primer 5-ATGGCCGCTTTAATGGCACG -3, reverse primer 5-GCCCGTTGTACGTTGTGTG -3

Metacaspase-1: forward primer 5- AAA CGG GTC GAC ATT AAT GC-3, reverse primer 5- CGA GCA TGA GGA AAA GAT CA-3
