# Supplementary figures and images for: Apoptosis-like cell death in Leishmania donovani treated with KalsomeTM10, a new liposomal amphotericin B
Source: PLoS One. 2017 Feb 7;12(2):e0171306. doi: 10.1371/journal.pone.0171306 (PMC5295687; doi:10.1371/journal.pone.0171306)

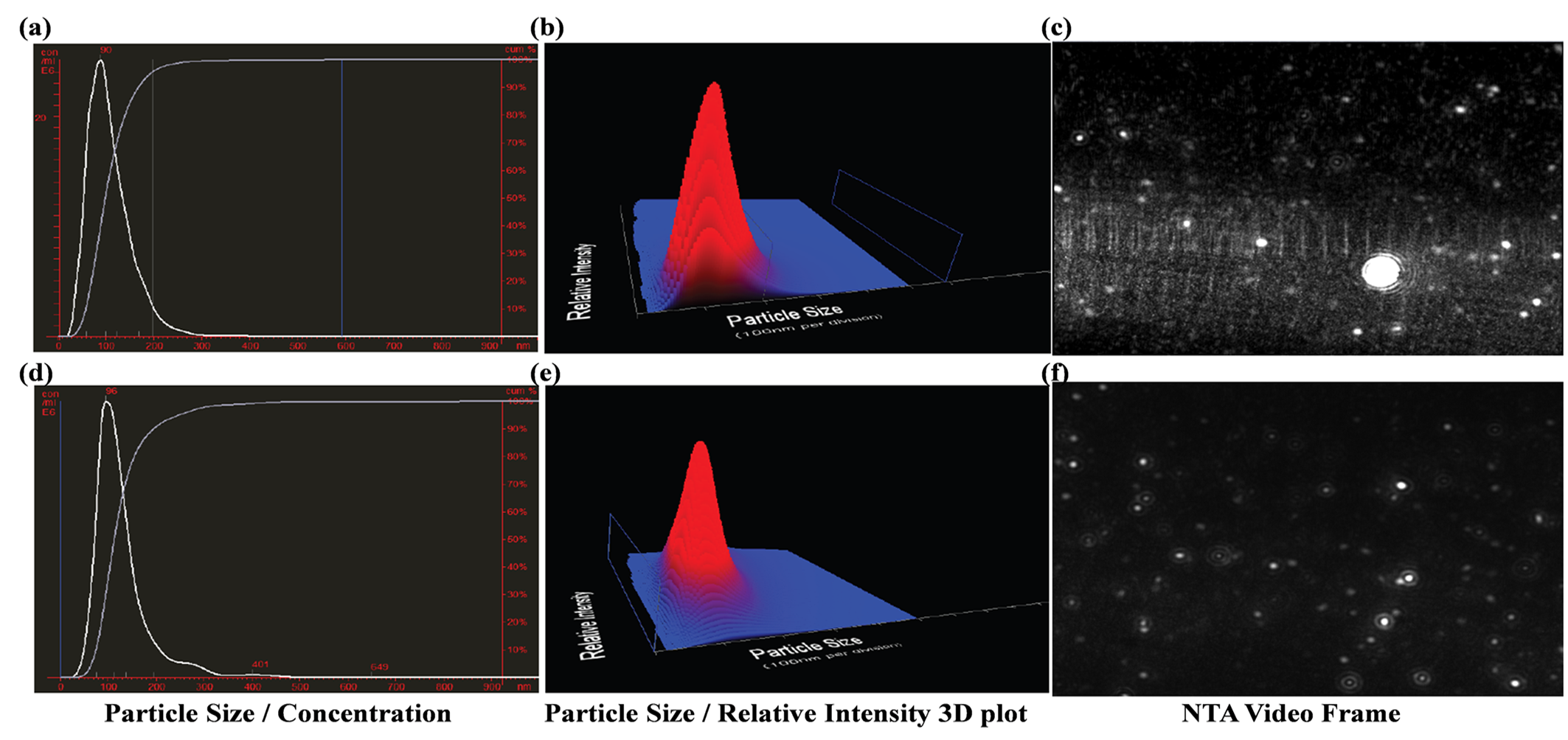

Supplement: S1 Fig — KalsomeTM10 (A-C) and Ambisome (D-F) (left panels) with the corresponding NTA video frame (right panels) and 3D graph (size vs. intensity vs. concentration; (middle panels). (TIF) [file pone.0171306.s002.tif]

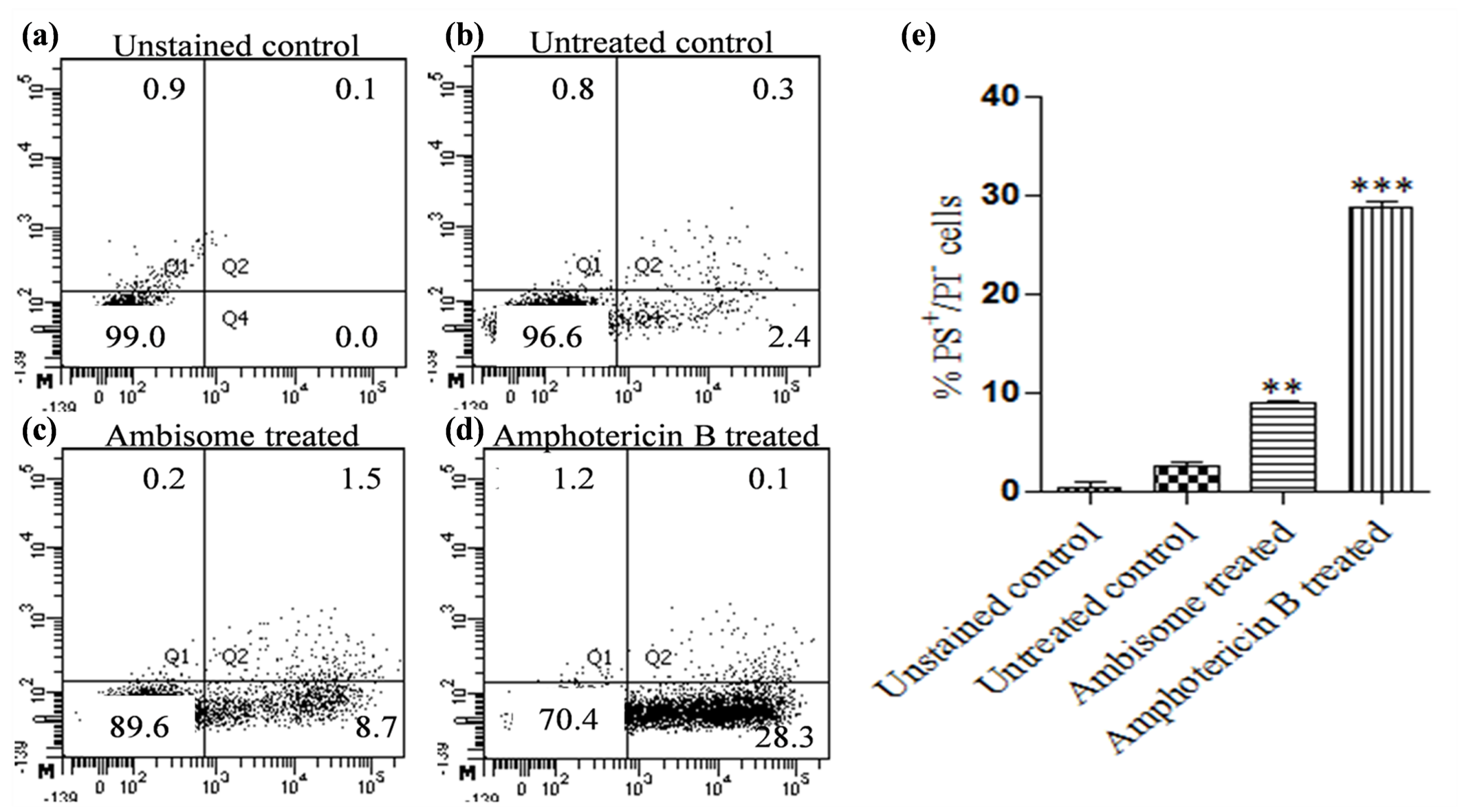

Supplement: S2 Fig — Promastigotes, untreated (a and b) and treated with Ambisome at 7.5 μg/ml (c) and amphotericin B at 0.25 μg/ml (d) for 1 h, were co stained with annexin V-FITC and PI, and analysed by flow cytometry. The dot plots are representative of two independent experiments (e) Bar graphs representing mean % PS+/PI− cells. **P<0.001, ***P<0.0001. (TIF) [file pone.0171306.s003.tif]

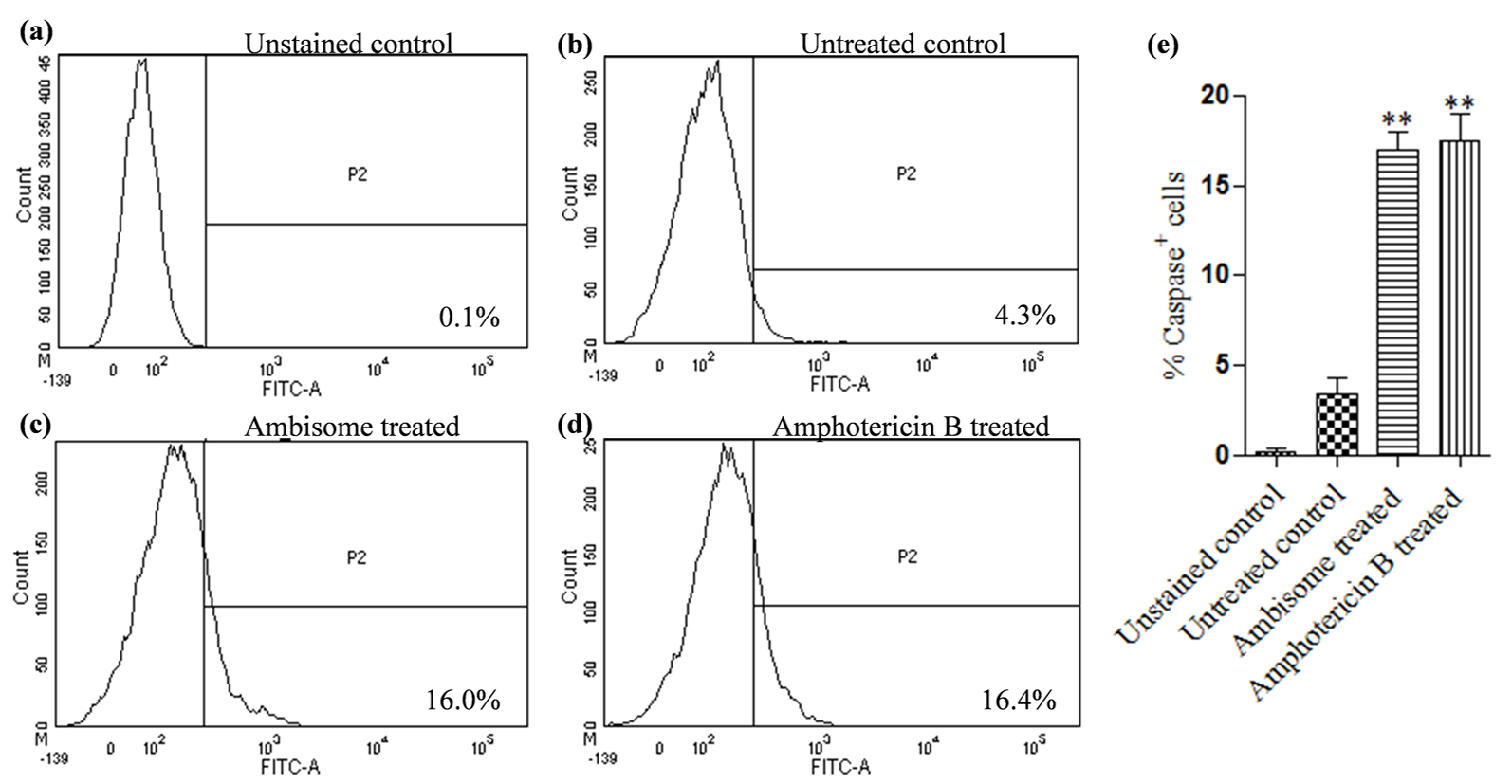

Supplement: S3 Fig — Promastigotes were either left untreated (a and b) or treated with Ambisome at 7.5 μg/ml (c) and amphotericin B at 0.25 μg/ml (d) for 1 h. Caspase positive cells were analysed through flow cytometry and plotted as histograms which are representative of two independent experiments. (e) Bar graphs representing mean % caspase+ cells. ***P<0.0001. (TIF) [file pone.0171306.s004.tif]

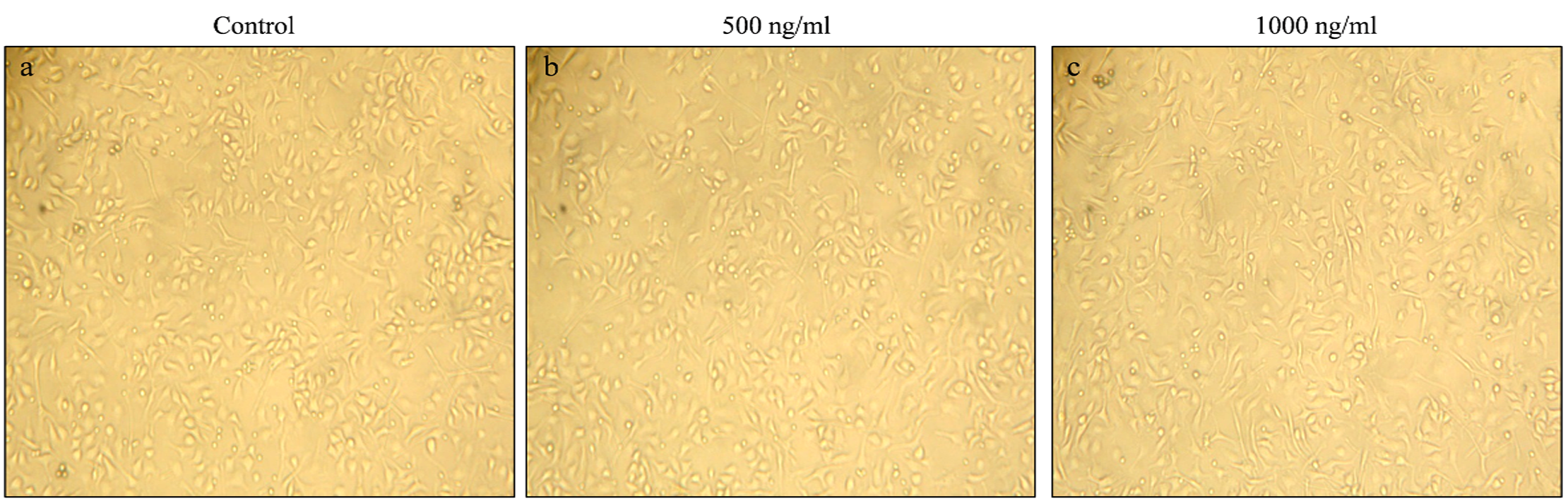

Supplement: S4 Fig — Macrophages, infected with promastigotes, were either untreated (A) or treated with 500 ng/ml (B) and 1000 ng/ml of KalsomeTM10 for 72 h post infection. The images of these cells were captured under a light microscope. (TIF) [file pone.0171306.s005.tif]

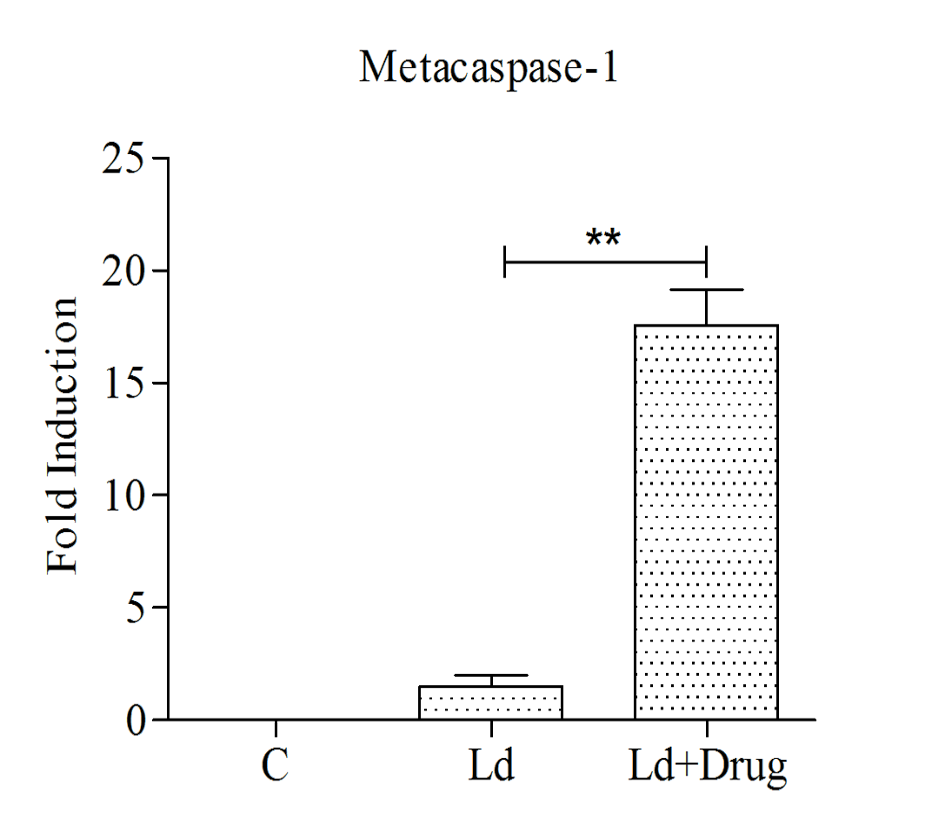

Supplement: S5 Fig — RAW 264.7 macrophages infected with promastigotes of L. donovani were either untreated or treated with 500 ng/ml of KalsomeTM10 for 2 h. The change in gene expression was determined through Real-time PCR using specific primers for L. donovani metacaspase-1and GAPDH (used as internal control). **P<0.05. (TIF) [file pone.0171306.s006.tif]
